# E2 protein is the major determinant of specificity at the human papillomavirus origin of replication

**DOI:** 10.1371/journal.pone.0224334

**Published:** 2019-10-23

**Authors:** Airiin Laaneväli, Mart Ustav, Ene Ustav, Marko Piirsoo

**Affiliations:** 1 Institute of Technology, University of Tartu, Tartu, Tartumaa, Estonia; 2 Icosagen Cell Factory Ltd., Õssu, Kambja, Tartumaa, Estonia; 3 Estonian Academy of Sciences, Tallinn, Harjumaa, Estonia; Consejo Superior de Investigaciones Cientificas, SPAIN

## Abstract

The replication of human papillomavirus (HPV) genomes requires E1 and E2 proteins as the viral *trans*-factors and the replication origin, located in the URR, as a *cis*-element. The minimal requirements for an HPV replication origin vary among different virus types but always include one or more binding sites for the E2 protein. The requirements for an E1 binding site seem to vary among different HPV genera, with *alpha*-HPV11 and -18 minimal origins able to replicate without E1 binding site in contrast to *beta*-HPV8. In the present article, we analysed the sequence requirements for the *beta*-HPV5 minimal origin of replication. We show that the HPV5 URR is able to replicate in U2OS cells without the sequence proposed as an E1 binding site, albeit at lower levels than wt URR, given that three E2 binding sites are intact and both viral replication proteins are present. The lack of an absolute requirement of the E1 binding site for the origin of replication of HPV5 led us to analyse whether the viral E1 and E2 proteins from other HPV types are competent to support replication from this origin. Surprisingly, the E1 and E2 proteins from *beta*-HPV types support replication from the origin in contrast to proteins from *alpha*-HPV types 11, -16, or -18. Furthermore, the replication proteins E1 and E2 of these *alpha*-HPV types are unable to support the replication of HPV5 URR, even if the E1 binding site is intact. In light of these results, we performed a detailed analysis of the ability of different combinations of E1 and E2 proteins from various *alpha-* and *beta-*HPV types to support the replication of URR sequences from the respective HPV types in the U2OS cell line.

## Introduction

Papillomaviruses (PVs) are widely spread oncogenic small DNA viruses that infect keratinocytes of cutaneous and mucosal epithelial tissues. More than 300 different papillomavirus types from humans (over 200 types) and other mammals, birds and reptiles have been described and completely sequenced to date (PaVe:Papillomavirus Episteme https://pave.niaid.nih.gov/) [1]. Human papillomaviruses (HPVs) are divided into five genera: *alpha-*, *beta-*, *gamma-*, *mu-*, and *nu-*papillomaviruses [2] [3].

To date, the best-studied group of HPVs is the mucosal epithelium infecting *alpha-*papillomaviruses (*alpha*-PVs). Several high-risk subtypes of these viruses (for instance, HPV16, HPV18, HPV31) cause cervical [4] [5] and other anogenital cancers [6] and are implicated in the development of head and neck cancers [7].

In recent years, the cutaneous epithelium infecting *beta-*papillomaviruses (*beta*-PVs) have garnered significant attention due to their involvement in cutaneous *squamous cell carcinoma* (SCC) in *epidermodysplasia verruciformis* (EV) patients and their role in the development of non-melanoma skin cancer (NMSC), especially in immunocompromised and immunosuppressed patients (reviewed in [8]). HPV5 and -8 have been detected in 90% of cutaneous SCCs of EV patients, and their E6 proteins possess cell-transforming potential *in vitro*. These two HPVs are considered high-risk *beta*-PVs [9] [10] [11] [12] [13] [14] [15].

Replication of the HPV genomes is largely carried out by host cell factors. Viral elements necessary and sufficient for HPV DNA replication include a *cis*-acting origin of replication and *trans*-acting E1 and E2 proteins (reviewed in [16]). E1 is the primary replication protein that acts as a helicase that specifically binds, melts and unwinds the viral replication origin to allow access of the cellular replication proteins. E2 is the loading protein that interacts with E1 and helps it to bind to the viral origin. The replication origin is located in the non-coding part of the viral genome called the Upstream Regulatory Region (URR). URRs of different HPV types vary in length and structure, starting from 478 base pairs in the case of HPV5 URR and reaching 953 base pairs in the case of HPV31 [17].

The minimal origin of replication of PVs is defined as the shortest *cis*-sequence in URR, which still supports replication under permissive conditions in the presence of *trans-* factors. The minimal origin of replication of PVs is most thoroughly characterized for bovine papillomavirus type 1 (BPV1). It has been shown that the BPV1 minimal replication origin is 90 base pairs long and includes one E1 binding site (E1BS), two E2 binding sites (E2BSs) and an A/T-rich region [18]. In the case of HPVs, it has been shown that the HPV11 and -18 minimal replication origins can function without E1 binding sites if they still include two high-affinity E2 binding sites alone or one E2BS plus an A/T-rich region [19] [20] [21].

Different organization of *alpha-* and *beta-*HPV URRs (schematically depicted in S1A Fig) might result in the differential requirements for the minimal *cis*-sequences for replication in these genera. The nature of the minimal origin of replication of cutaneous epithelium infecting *beta*-papillomaviruses has remained less thoroughly understood. The minimal replication origin has been described only for HPV8 from *β-*HPVs [22], which together with HPV5 represents the high-risk *beta-*PV types [10] [23]. The smallest URR fragment necessary and sufficient to fulfil the replication origin function in HPV8 consists of a 65-bp DNA fragment containing two sequence elements, E1BS and M29. M29 contains an unconventional E2BS, which is conserved among most EV-HPVs [24] [22].

We have developed a cellular assay system for studying HPV genome replication based on the human osteosarcoma U2OS cell line, which has the capability to support the transient, stable, and late amplification replication of both cutaneous and mucosal HPV genomes [25].

In the present article, we describe the minimal origin of replication of cutaneous epithelium infecting HPV5, which replicates in U2OS cells [25][26]. We show that surprisingly, the sequence element proposed to function as the E1 binding site but not the E1 protein itself is dispensable for the HPV5 minimal replication origin. We further show that the replication of E1 binding site minus origin requires *beta*-papillomavirus E2 protein and is not working if E2 proteins from *alpha*-PVs are present. We also show that neither HPV5, HPV8 nor HPV38 URR sequences replicate in the presence of *alpha*-papillomavirus E2 proteins, whereas replication of *α*-papillomavirus URRs occurs in the presence of *β*-PVs E2 proteins. Taken together, our data show that differences exist in the minimally required *cis*-sequences of replication origins of *alpha-* and *beta-*HPVs. Additionally, the ability of viral replication proteins from different HPV genera varies to support replication of these *cis*-sequences.

## Materials and methods

### Plasmids

pUC18-based HPV11, -16 and -18 URR plasmids have been described previously [27]. HPV5, -8 and -38 URR plasmids were created by amplifying the URR sequences (nt. 7468–199 in HPV5; nt. 7396–195 in HPV8; nt. 7194–199 in HPV38) from the respective genomes in the pBR322 plasmid by PCR with primers containing the *XbaI* or *EcoRI* site. The PCR fragments were cloned into the multicloning site of the pUC18 plasmid between the *XbaI* and *EcoRI* sites. Deletion mutants URR I (nt. 7467–23), -II (nt. 8–199) and -III (nt. 8–133) for HPV5 URR were created in a similar manner. Deletion mutants URR IV (nt. 7738–7748) and -V (nt. 20–85) were generated by cloning the respective sequences into the *HincII* site of pUC18 as a double-stranded oligonucleotide. All numerations of nucleotides correspond to the reference genomes deposited in the papillomavirus episteme (https://pave.niaid.nih.gov/). All constructs were verified by sequencing. Sequences of the oligonucleotides used are shown in the S1 Table.

HPV11, -16 and -18 wild-type E1 and E2 and HPV18 E1 mutant (K237A) expression vectors have been described previously [27][28]. Expression vectors coding for HPV5, -8 and -38 E2 proteins were generated by amplifying the E2 coding sequence using PCR with respective primers containing the *BamHI* and *HindIII* (HPV5 and -8) or *HindIII* and *XmaI* sites (HPV38). The PCR fragment was cloned into the multicloning site of eukaryotic expression vector pQM-NTag/Ai+ (Quattromed Ltd) between *BamHI* and *HindIII* sites (HPV5 and -8) or *HindIII* and *XmaI* sites (HPV38). The *β*-globin intron was removed from the plasmid with restriction enzymes *HindIII* and *BglII* (HPV5 and HPV8) or *Cfr9I* and *BglII* (HPV38). An HPV5 E1 expression vector was generated in two steps. First, the pQMNTAiHPV5E1 plasmid was generated by amplifying the E1 ORF sequence (nt 961–2781) with PCR and cloning the fragment into the pQM-NTag/Ai vector between the *XmaI* and *XbaI* restriction sites. Second, a 1088-bp HPV5 DNA (nt 201–1251, containing the E6, E7 and the 5’ part of the E1 ORF) was synthesized. The following changes were made in the synthetic HPV sequence: initiation codons for E6 and E7 were mutated; major donor splice site (AGGT) at the beginning of E1 ORFs was disrupted by inserting influenza haemagglutinin epitope tag (HA) in-frame into the E1 coding sequence. The presence of E6 and E7 sequences in front of E1 ORF improve the translation efficiency of E1 protein. This synthetic DNA was cloned into the pQMNTAiHPV5E1 plasmid between the *XmaI* and *BoxI* sites, resulting in the pQMNTAiHPV5E1 expression vector. The HPV8 E1 expression vector was generated by separately amplifying HPV8 E6 and E7 ORFs and the E1 ORF (nt 196–2762) of the HPV8 genome. The PCR products of E6 and E7 ORFs were cleaved with *XmaI* and *Esp31I*, and the E1 PCR product was cleaved with *XbaI* and *Esp31I*. The amplified fragments were cloned into the pQM-NTAi vector between the *Xma1* and *Xba1* sites. The E6 start codon was mutated in the 5’ primer used in PCR. The HA-tag was inserted into the E1 donor splice site, and the E7 ATG was mutated by PCR-mediated mutagenesis.

### Transient replication assays

U2OS cells were grown in Iscove`s modified Dulbecco`s medium (IMDM) supplemented with 10% foetal calf serum, 100 U/ml penicillin and 0.1 mg/ml streptomycin. The cells were transfected using electroporation at 220 V and capacitance set to 975 μF using a Bio-Rad Gene Pulser II apparatus supplied with a capacitance extender. Cells were transfected with 500 ng of ori plasmid combined with 100 ng of E1 expression vector and 250 ng of E2 expression vector. Five million cells were used per transfection. The cells were grown on 100 mm Petri dishes, and DNA was extracted 24, 48, 72 and 96 h post-transfection. Low-molecular-weight DNA was extracted from the cells by the modified Hirt lysis procedure [29], and total DNA was extracted by the standard SDS-proteinase K extraction method. DNA samples were digested with an enzyme *ScaI*, which linearizes ori plasmid and with *DpnI* to remove the bacterially methylated non-replicated DNA. In the case of Hirt extraction, half of the extracted DNA was used for analysis, and in the case of total DNA, five micrograms were used. Digested DNA was size-fractionated in agarose gels, transferred to a nylon filter and analysed by Southern blotting (SB). Linearized pUC18 plasmid was used as a probe for SB. All SB assays were performed at least three times, and representative images are shown. SB signals corresponding to the replicated DNA were quantified using ImageQuant software.

### Western blot analysis

U2OS cells (5 million cells) were transfected with 100 ng of E1 expression vectors with or without 250 ng of E2 expression vectors. Protein extracts were made 48 h after transfection, either by lysing the cells directly in Laemmli buffer or by making RIPA extracts. Protein extracts were resolved in 10% SDS-PAGE, followed by transfer to a PVDF membrane. Membranes were probed with an HRP-conjugated antibody 3F10 (1:10 000; Roche) or 16B12 (1:1000 or 1:750; BioLegend), raised against the HA-tag, to detect E1 levels and GAPDH antibody (1:10 000; Sigma) to estimate loaded protein amounts. After washing, the filters were incubated with GAM-HRP secondary antibody (1:10 000; LabAs Ltd). Signals were visualized using an ECL^TM^ Kit (Amersham Pharmacia Biotech). At least three independent western blot analyses were performed and a representative image is shown.

## Results

### HPV5 URR can replicate without the putative E1 binding site

**Fig 1:**
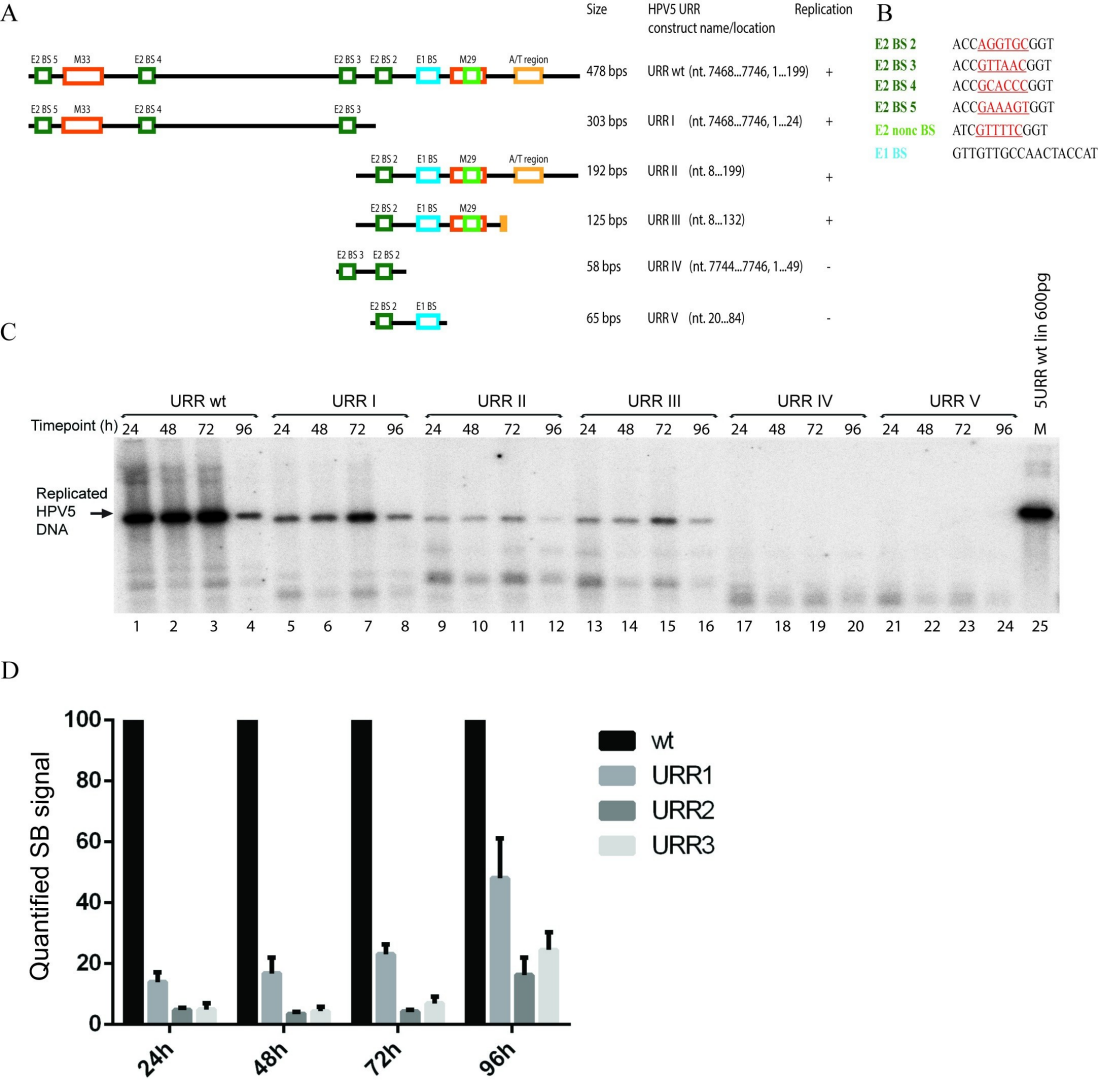
DNA replication is triggered from HPV5 origin without a binding site for E1 protein. (A) Schematic representation of HPV5 URR fragments used in transient replication assays in U2OS cells. The behaviour of the respective construct in the replication assay is shown on the right. Green boxes represent E2 binding sites, blue boxes represent the E1 binding site, orange boxes represent the M29 region overlapping with a non-consensus E2 binding site and the M33 region, and yellow boxes represent an A/T-rich region. (B) Sequences of E2 and E1 binding sites in HPV5 URR. E2 nonc BS refers to non-consensus E2 binding site found in the M29 region of the HPV5 URR. (C) Transient replication analysis of the HPV5 URR constructs depicted in A. U2OS cells were transfected with 500 ng of HPV5 URR plasmids together with 250 ng of HPV5 E2 and 100 ng of HPV5 E1 expression vectors. Twenty-four, 48, 72 and 96 h after transfection, low-molecular-weight DNA was isolated and digested with *DpnI* to remove input DNA and an enzyme linearizing the construct (*ScaI*). Replication was analysed by Southern blotting (SB). (D) SB signals from three independent experiments were quantified and set as 100% for the wt URR construct. Data are presented as an average mean +/- standard deviation (SD).

To determine the sequence requirements for a functional HPV5 origin of replication, we made a series of deletions in the URR. HPV5 URR and the deletion mutants used in this study are depicted in Fig 1A. The HPV5 genome has five E2 binding sites, four of which are situated in the URR (binding sites 2–5). In addition, a non-conventional E2 binding site has been mapped (inside the M29 region, which is conserved in EV papillomaviruses) to the close vicinity of the putative E1 binding site [22]. To analyse which regions of the URR are necessary for HPV5 DNA replication, we first divided the entire URR region into two half-parts (Fig 1A, URR I and URR II) and cloned the respective DNA fragments into the pUC18 vector. The URR I construct contains three E2 binding sites (E2BS 3–5) and the M33 region, and URR II contains one high-affinity E2 binding site (E2BS 2), a putative E1 binding site, an M29 region with an atypical E2 binding site, and an A/T-rich region. Sequences of all E2 binding sites and a putative E1 binding site are depicted in Fig 1B. These constructs were co-transfected into U2OS cells with HPV5 E2 and E1 expression vectors, and low-molecular-weight DNA was extracted at different time points post-transfection. Replication was analysed by SB. Both constructs were able to replicate in U2OS cells in a transient replication assay, albeit less efficiently than the whole URR (compare lanes 1–4 with 5–8 and 9–12 in Fig 1C). Quantification of the SB results showed that the replication efficiency of the URR I construct was 14% at 24 hours, 17% at 48 hours, 24% at 72 hours, and 48% at 96 hours after transfection, as compared to the wt URR construct. The URR II construct replicated even less efficiently, with 16% compared to the wt URR at 96 hours after transfection (Fig 1D).

The URR II construct, which contains one E2 binding site, a putative E1 binding site, an M29 region and an A/T-rich region, was able to replicate in U2OS cells. To investigate whether all of these elements are absolutely necessary *cis*-elements for replication, we generated additional deletion mutants URR III (containing one high-affinity E2 binding site, an E1 binding site, an M29 region, and no A/T-rich region), URR IV (containing two high-affinity E2 binding sites and no E1 binding site) and URR V (containing one high-affinity E2 binding site and an E1 binding site). Our analysis showed that in addition to the HPV5 wt URR, URR I and URR II constructs, URR III was able to replicate if E1 and E2 proteins were provided in *trans* (Fig 1C, lanes 13–16), while URR IV and V were not (Fig 1C, lanes 17–24). The replication efficiency of the URR III construct was comparable to the URRII construct (Fig 1C and 1D).

**Fig 2:**
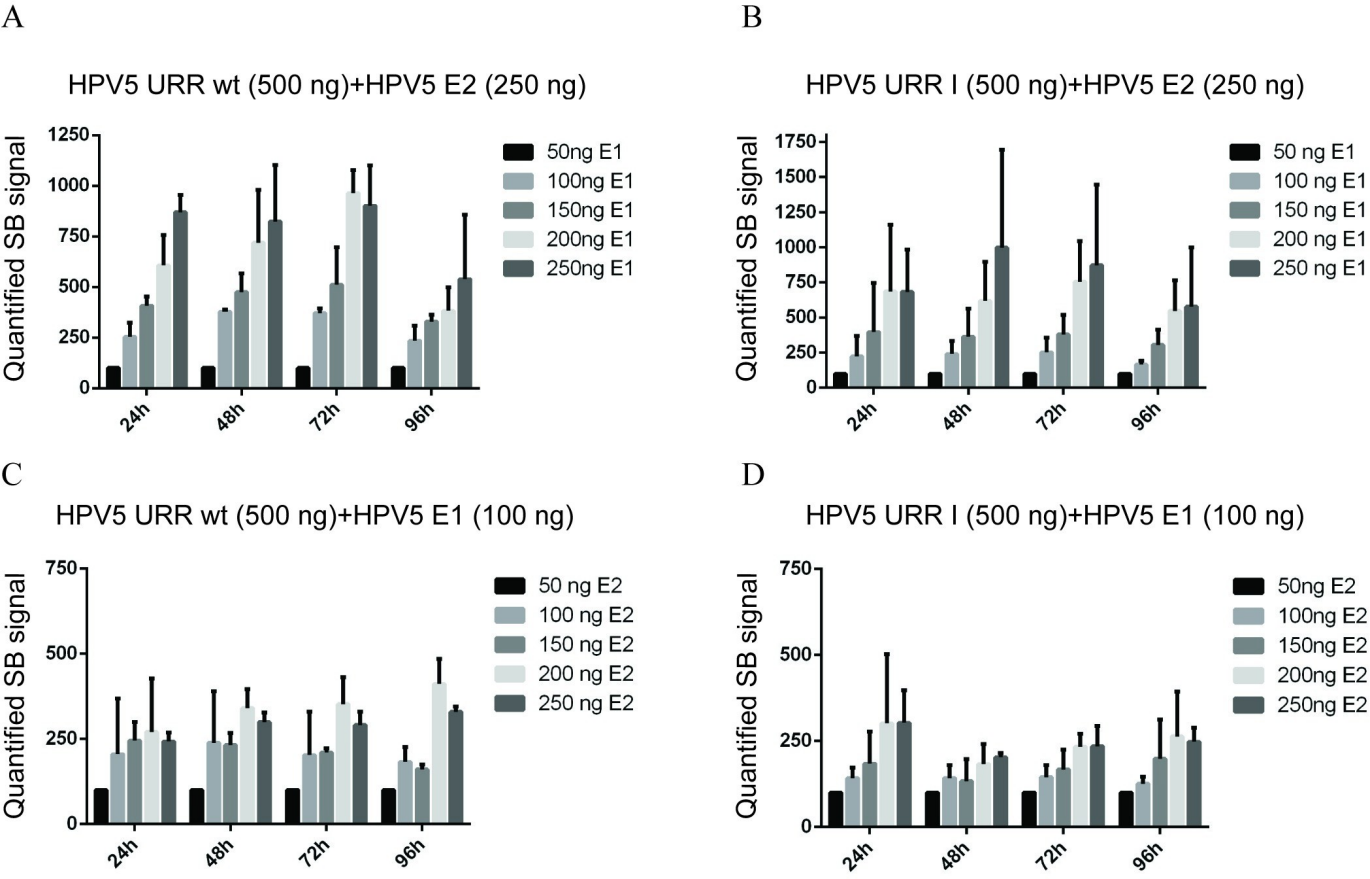
Influence of the amounts of E1 and E2 proteins on the replication efficiencies of the HPV5 wt URR and URR I constructs. Quantification of the transient replication signals of the HPV5 wt URR (A and C) and URR I (B and D) constructs in the presence of fixed amounts of E2 expression vector combined with increasing amounts of E1 expression vector (A and B) or fixed amounts of E1 expression vector combined with increasing amounts of E2 expression vector (C and D). SB signals from three independent experiments were quantified and set as 100% for smallest amount (50 ng) of variable expression vector (E1 or E2) at each timepoint. Data are presented as an average mean +/- SD.

Given that the HPV5 URR I construct lacks an E1 binding site, as opposed to the wt URR, one could hypothesize that the necessary levels of viral proteins to achieve maximum levels of replication differ if these different origins are used. To control this possibility, we co-transfected HPV5 wt URR or URR I plasmids together with fixed amounts of HPV5 E2 expression vector and increasing amounts of HPV5 E1 expression vector (from 10 ng to 250 ng) into the U2OS cells. At different time points post-transfection, the total DNA was extracted and analysed by SB (S2A and S2B Fig). Replication signals from three independent experiments were quantified and are shown in Fig 2A and 2B.

The replication signal of wt HPV5 origin increased with increasing amounts of E1 expression vector (Fig 2A and S2A Fig). Similarly, replication of HPV5 E1BS minus origin (URR I) was also dependent on the concentration of the E1 expression vector, although that origin does not contain the E1 binding site but has only three E2 binding sites (Fig 2B and S2B Fig). The replication level increased as the amount of expression plasmid of E1 increased but was still maintained at a lower level than the replication of the wild-type origin plasmid (S2B Fig, lanes 25–28).

As it appeared that replication of HPV5 E1BS minus origin was still dependent on the concentration of E1 protein, although it does not possess any E1 binding sites, we also wanted to know how much that origin of replication was affected by the levels of the other viral replication protein E2, as there are three E2 binding sites in this origin construct.

HPV5 wt URR or URR I plasmids were co-transfected together with a fixed amount of HPV5 E1 expression vector and increasing amounts of HPV5 E2 expression vector (from 10 ng to 250 ng) into the U2OS cells. Replication of wild-type HPV5 origin increased with increasing amounts of E2 expression vector, reaching a plateau at 200 ng (Fig 2C and S2C Fig). Similar results were obtained using HPV5 E1BS minus origin (Fig 2D and S2D Fig).

Taken together, our results show that HPV5 origin can replicate under permissive conditions without E1 binding sites, given that at least 3 E2 binding sites in the URR region are present (Fig 1C, lanes 5–8). In this study, we also identified that another short sequence from the HPV5 URR, containing one high-affinity E2 binding site, an E1 binding site and an M29 sequence element (URR III), functions as the HPV5 origin of replication.

### E1 and E2 proteins from other *beta*-papillomavirus types support replication of HPV5 E1 binding site minus origin

Next, we examined whether replication of the HPV5 origin lacking the E1 binding site (URR I) occurs only by HPV5 E1 and E2 proteins, as the type-specific *trans*-elements or viral replication proteins from other *beta*-PV types are also able to support replication of HPV5 E1BS minus origin.

URR I or HPV5 wild-type origin containing plasmids were co-transfected together with HPV5 or HPV8 E1 and E2 expression vectors into the U2OS cells. Total DNA was extracted 24–96 h after the transfection, digested with appropriate restriction enzymes, and resolved by agarose gel electrophoresis, and replication was analysed by SB.

**Fig 3:**
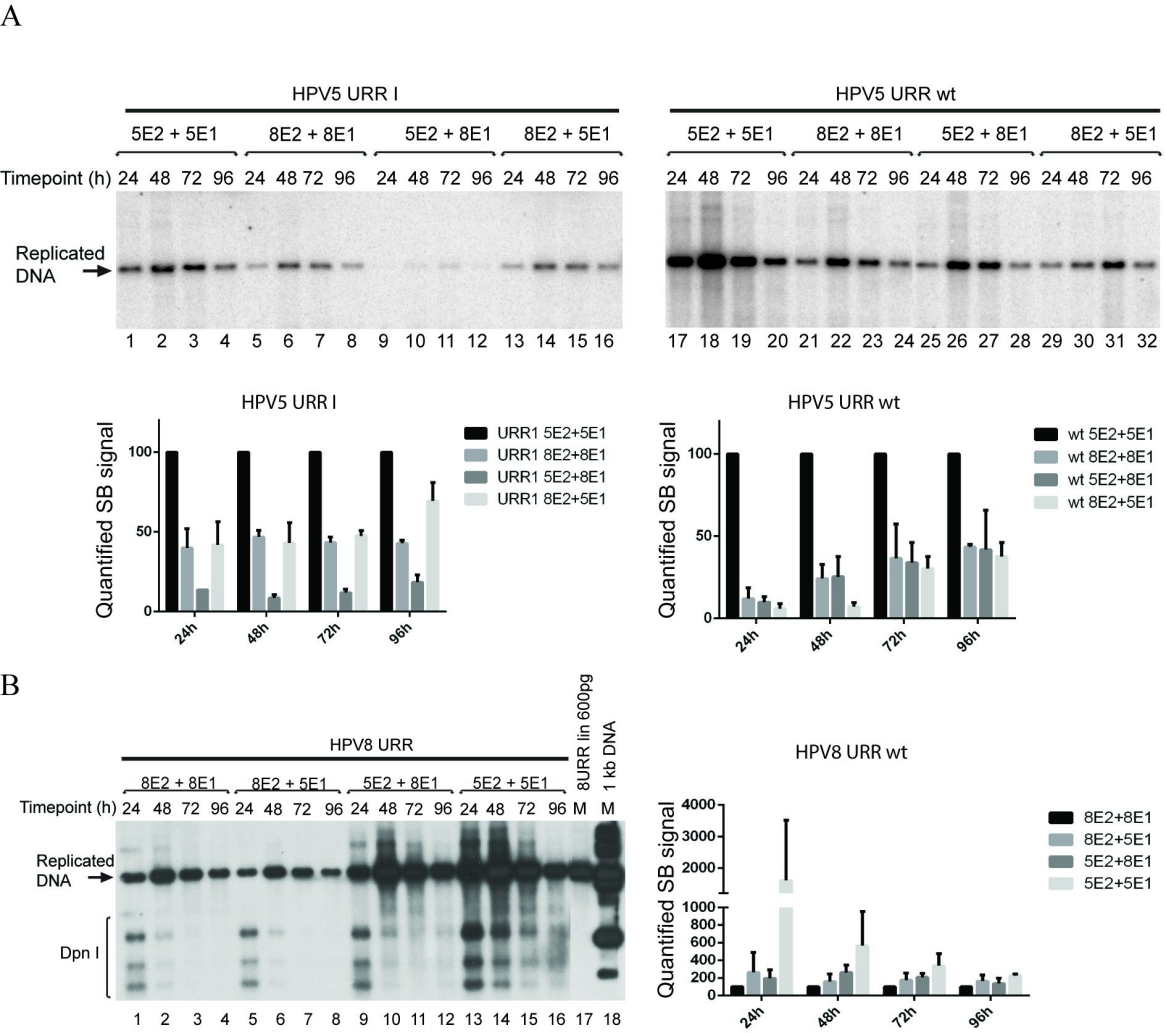
E1 and E2 proteins from HPV types 5 and 8 can be combined to support replication of HPV5 and 8 origins. (A) Replication of HPV5 wild-type and E1BS minus origin (URR I) in the presence of a combination of HPV5 and HPV8 E1 and E2 proteins. U2OS cells were co-transfected with 500 ng HPV5 URR I (lanes 1 to 16) or HPV5 URR wt (lanes 17 to 32) plasmids together with 100 ng HPV5 or HPV8 E1 expression vector and 250 ng HPV5 or HPV8 E2 expression vector. Total DNA was extracted at the indicated time-points after transfection. DNA was digested with *DpnI* to remove input DNA and an enzyme linearizing the construct (*ScaI*), resolved in agarose gel, and replication was analysed by SB (upper panel). Quantification of the SB signals is shown under the SB images. SB signals from three independent experiments were quantified and set as 100% for the HPV5 E1 and E2 combination. Data are presented as an average mean +/- SD. (B) Replication of HPV8 origin in the presence of a combination of HPV5 and HPV8 E1 and E2 proteins. U2OS cells were co-transfected with 500 ng HPV8 URR together with 100 ng HPV5 or HPV8 E1 expression vector and 250 ng HPV5 or HPV8 E2 expression vector. Total DNA was extracted at the indicated time-points after transfection. DNA was digested with *DpnI* to remove input DNA and an enzyme *ScaI* linearizing the construct, resolved in agarose gel, and replication was analysed by SB (left panel). Quantification of the SB signals is shown on right panel. SB signals from three independent experiments were quantified and set as 100% for the HPV8 E1 and E2 combination. Data are presented as an average mean +/- SD.

Our results showed that HPV5 URR I origin was able to replicate in the presence of HPV8 E1 and E2 proteins (Fig 3A, lanes 5–8), similar to HPV5 wt origin (Fig 3A, lanes 21–24). HPV5 E1BS minus origin and wt origin were also able to replicate when a combination of E1 and E2 proteins derived from different *beta*-HPV types were used–HPV5 E2 in combination with HPV8 E1 (Fig 3A, lanes 9–12 and 25–28) and HPV5 E1 in combination with HPV8 E2 (Fig 3A, lanes 13–16 and 29–32). Quantification of the results showed that the HPV8 E1 and E2 proteins, and the heterologous combinations of HPV replication proteins were all less potent triggerers of the replication of HPV5 wt URR and URR I origins than HPV5 E1 and E2 (Fig 3A, lower panels).

Next, we tested whether HPV8 URR can replicate in the presence of E1 and E2 proteins from HPV5. HPV8 origin containing plasmid pUC18-HPV8URR was co-transfected together with HPV5 or HPV8 E1 expression vectors and HPV5 or HPV8 E2 expression vector into the U2OS cells. The HPV8 wt origin plasmid was able to replicate in the presence of HPV5 E1 and E2 proteins (Fig 3B, lanes 13–16) and if viral replication proteins from different *beta* HPV types were mixed–HPV8 E2 and HPV5 E1 (Fig 3B, lanes 5–8), HPV5 E2 and HPV8 E1 (Fig 3B, lanes 9–12). Quantification of the results showed that HPV5 E1 and E2 proteins were the most potent *trans*- acting factors to support the replication of HPV8 URR (Fig 3B, right panel).

**Fig 4:**
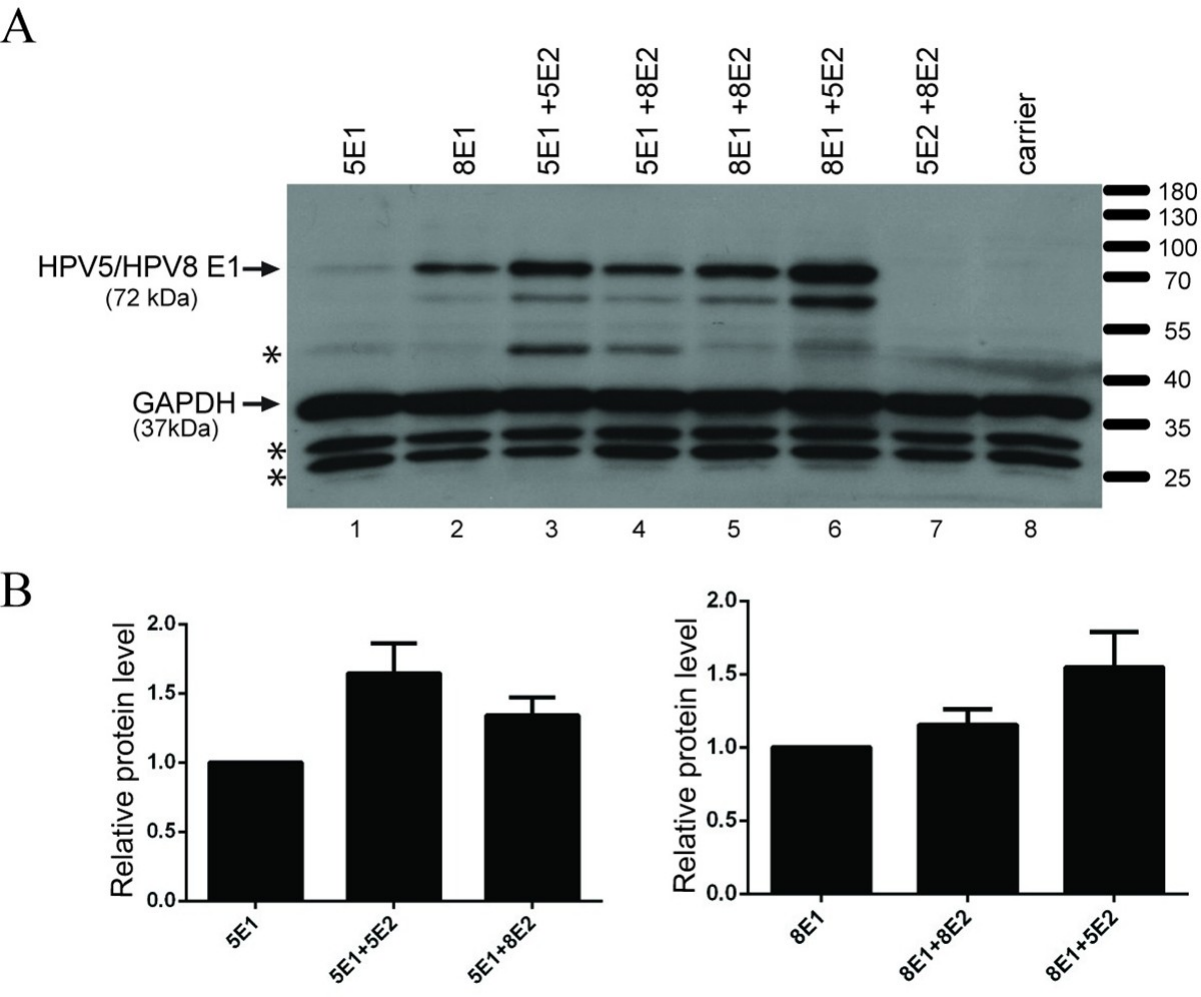
Expression levels of HPV5 and HPV8 E1 proteins in U2OS cells. (A) Expression vectors coding for HPV5 and HPV8 E1 proteins were transfected into U2OS cells either alone (lanes 1 and 2) or together with HPV5 and HPV8 E2 expression constructs (lanes 3 to 6). Cells transfected with E2 vectors alone (lane 7) or with carrier (lane 8) were used as negative controls. Cells were grown on the 100-mm Petri dish and lysed with 300 μl of 2xLaemmli buffer 48 h after transfection and used to determine the levels of E1 proteins by Western blot analysis. Extracts were denatured, and 20 μl of protein was resolved by 10% SDS-PAGE. E1 levels were determined using an HRP-conjugated antibody 3F10 raised against the HA-tag. Levels of GAPDH were used as loading controls. Non-specific signals are shown by asterisks. (B) Quantification of the E1 protein levels depicted in (A). WB signals from three independent experiments were quantified and set as 1 for HPV5 E1 (left panel) and HPV8 E1 (right panel). Data are presented as an average mean +/- SD.

Our analysis showed that the replication signals for HPV5 and HPV8 URRs were higher in the presence of HPV5 E1 protein compared to HPV8 E1 protein (Fig 3). To determine whether this more efficient replication was due to the higher levels of HPV5 E1 protein, we carried out Western blot analysis of over-expressed E1 proteins. Equal amounts of expression vectors coding for HA-tagged HPV5 and HPV8 E1 proteins were transfected into U2OS cells either alone (Fig 4A, lanes 1 and 2) or together with the expression constructs for HPV5 and HPV8 E2 proteins (Fig 4A, lanes 3 to 6). Co-transfection with E2 expression vectors was carried out because it has been shown previously that E2 stabilizes E1 protein [30]. Cells were lysed 48 h after transfection, and protein levels were measured using an antibody against HA-tag. We can conclude from our Western blot analyses that HPV8 E1 is expressed at higher levels than HPV5 E1 (Fig 4A, lanes 1 and 2). Co-transfection with HPV5 and HPV8 E2 expression vectors led to higher steady-state E1 protein levels (Fig 4A, compare lanes 1 and 3; 2 and 5), whereas increase in the E1 protein levels was bigger if HPV5 E2 protein was present as compared to HPV8 E2 (Fig 4B).

### HPV5 E1BS minus origin can replicate in the presence of the HPV18 E1 mutant lacking the DNA binding activity

Since HPV5 origin was able to replicate without the E1 binding site, we investigated whether E1 DNA binding activity is a prerequisite for replication. We decided to use mutant HPV18 E1 protein K237A, which cannot bind DNA specifically. It has been shown previously that this mutation completely abrogates HPV-18 E1 DNA binding activity to its cognate site, but it is still functional to support replication of HPV18 URR [31] [28].

**Fig 5:**
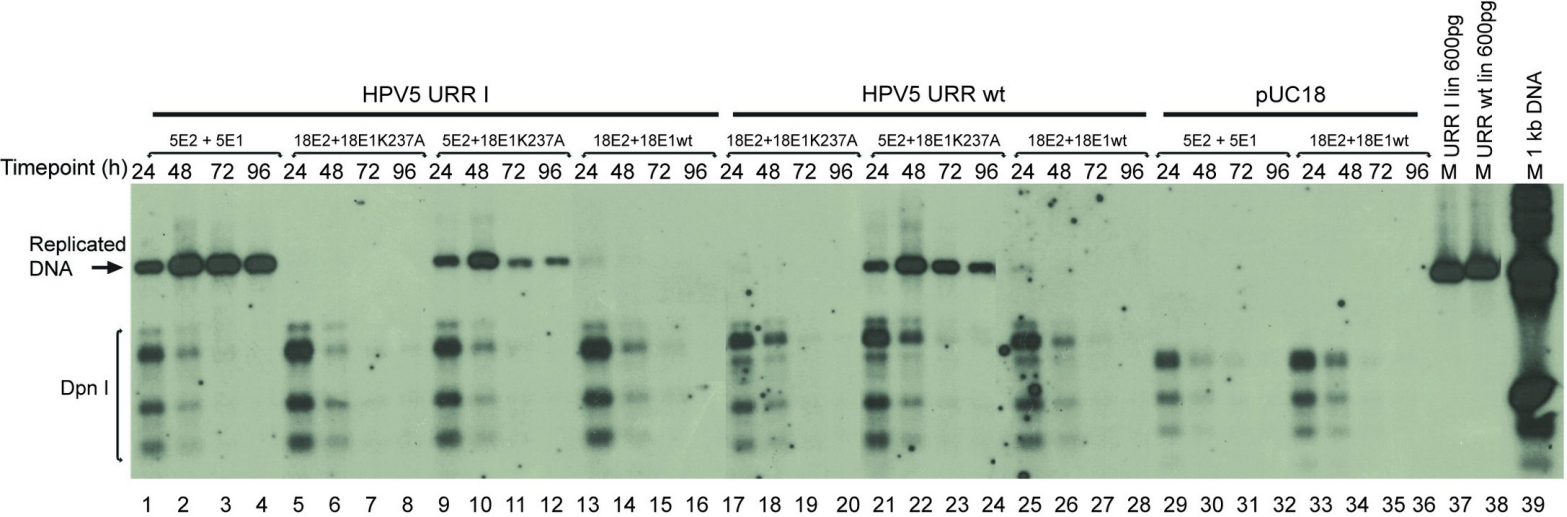
HPV5 E1BS minus origin is replicating with HPV18 E1 DNA binding deficient mutant protein in the presence of HPV5 E2 protein and not in the presence of HPV18 E2 protein. U2OS cells were co-transfected with 500 ng HPV5 URR I, HPV5 URR wt or pUC18 plasmids together with 100 ng HPV18 E1 K237A mutant or wt E1 expression vector and 250 ng HPV18 or HPV5 E2 expression vector. Total DNA was extracted at the indicated time points after transfection. DNA was digested with *DpnI* to remove input DNA and an enzyme (*ScaI*) linearizing the construct, resolved in agarose gel, and replication was analysed by SB.

HPV5 E1BS minus origin plasmid (URR I) was co-transfected together with HPV18 E1K237A mutant expression vector and HPV18 or HPV5 E2 expression vector. The results of these experiments showed that HPV5 origin was able to replicate, while there was neither E1 binding site in the replication origin nor functional DNA binding activity in the E1 protein (Fig 5, lanes 9–12) when HPV5 E2 expression was present in the system. The HPV5 wild-type origin also replicated in the presence of HPV18 E1K237A and HPV5 E2 proteins (Fig 5, lanes 21–24).

Given that the mutant E1 protein we used was derived from HPV18, we also tested whether HPV18 E1 and E2 proteins can support replication from HPV5 wt and HPV5 URR I origins. To our great surprise, we observed that neither HPV18 E1K237A nor wt E1 in combination with HPV18 E2 protein were able to support replication of HPV5 origins (Fig 5, lanes 5–8, 13–16, 17–20 and 25–28).

### HPV5 E1BS minus origin is not able to replicate in the presence of E2 proteins derived from *alpha*-papillomaviruses

**Fig 6:**
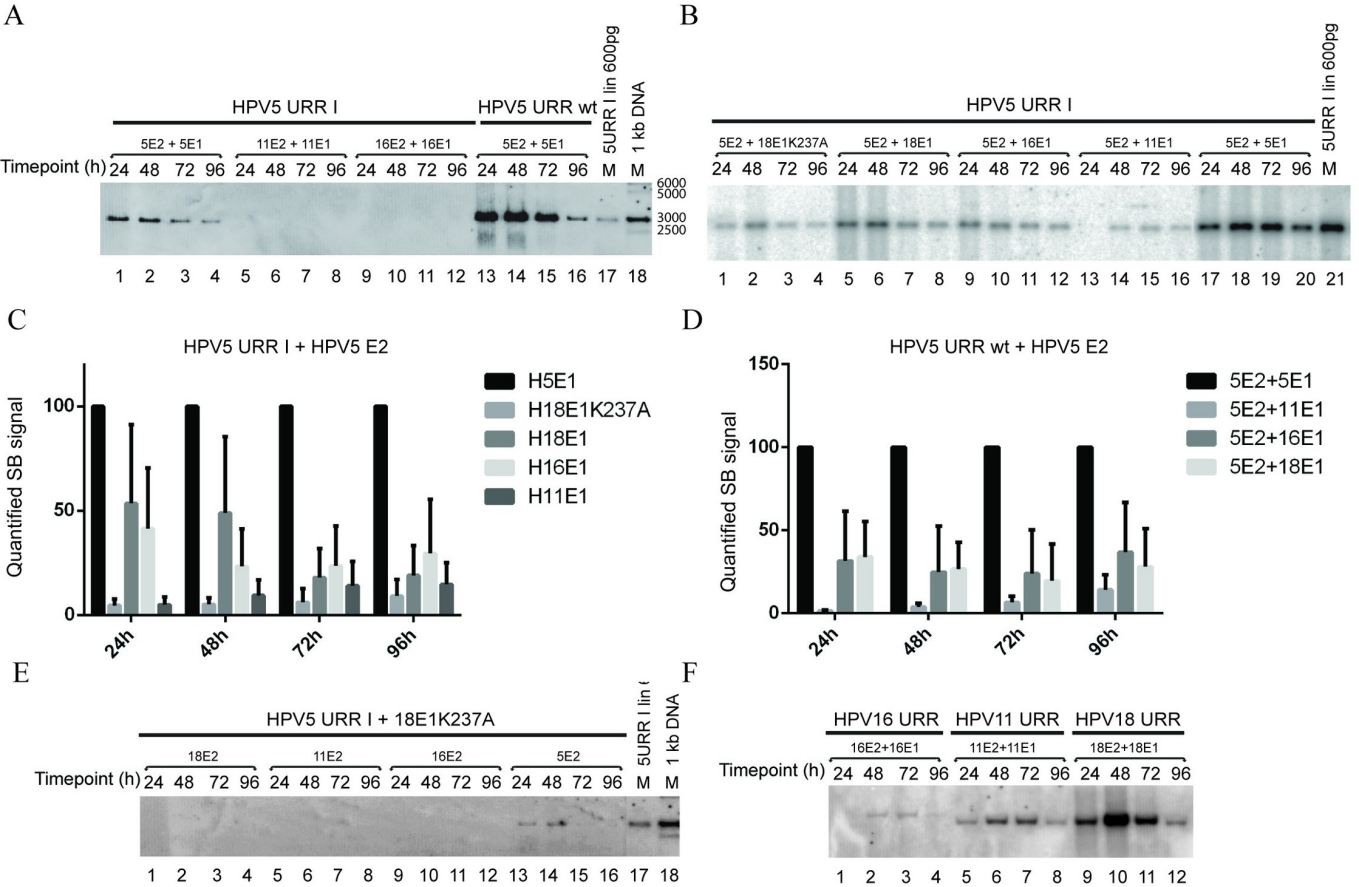
E2 proteins from *alpha* papillomaviruses do not support replication of HPV5 URR I. (A) Replication of HPV5 E1BS minus origin URR I in the presence of HPV11 and -16 viral replication proteins E1 and E2. U2OS cells were co-transfected with 500 ng HPV5 URR I (lanes 1 to 12) plasmid together with 100 ng HPV11 or HPV16 E1 expression vector and 250 ng HPV11 or HPV16 E2 expression vector. URR I or HPV5 URR wt in combination with HPV5 E1 and E2 expression vectors were used as positive controls (lanes 1–4 and 13–16). (B) Replication of HPV5 E1BS minus origin in the presence of HPV5 E2 and *alpha*-PVs E1 protein. U2OS cells were co-transfected with 500 ng HPV5 URR I plasmid together with 250 ng HPV5 E2 expression vector and 100 ng HPV5 (lanes 17–20), -11 (lanes 13–16), -16 (lanes 9–12) or -18 E1 wt (lanes 5–8) or HPV18 E1K237A (lanes 1–4) expression vector (upper panel). (C) Quantification of the SB signals of HPV5 URR I replicon combined with HPV5 E2 and *alpha*-PVs E1 expression vectors. (D) Quantification of the SB signals of HPV5 URR wt replicon combined with HPV5 E2 and *alpha*-PVs E1 expression vectors. SB signals from three independent experiments were quantified in (C) and (D) and set as 100% for the HPV5 E1 and E2 combination. Data are presented as an average mean +/- SD. (E) Replication of HPV5 E1BS minus origin in the presence of HPV18 E1K237A expression vector in combination with HPV5 (lanes 13–16), -11 (lanes 5–8), -16 (lanes 9–12) and -18 (lanes 1–4) E2 expression vectors. (F) Replication of URRs from HPV types 11 (lanes 5–8), -16 (lanes 1–4) and -18 (lanes 9–12) in combination with the viral *trans*-factors derived from the same virus type. HPV plasmids (500 ng) were transfected together with 100 ng E1 and 250 ng E2 expression vectors of the respective virus types into U2OS cells. Total DNA was extracted at the indicated time points after transfection for all panels. DNA was digested with *DpnI* to remove input DNA, and an enzyme *(ScaI*) linearized the construct, which was resolved in agarose gel, and replication was analysed by SB.

Since HPV18 E1 and E2 proteins were not able to support replication from HPV5 origin, we asked whether this finding is part of a wider phenomenon and can also be attributed to other *alpha-*papillomaviruses. We co-transfected HPV5 URR I together with HPV11 or -16 E1 and E2 expression vectors and analysed the replication competence of the origin-bearing plasmid. As shown in Fig 6, no replication was observed if expression vectors coding for E1 and E2 from HPV11 or -16 were used (Fig 6A, lanes 5–12).

Next, as a combination of HPV5 E2 and HPV18 E1 proteins supported replication from HPV5 URR I origin (Fig 5), we asked whether E1 proteins from other *alpha*-HPV types could behave in a similar manner. We co-transfected HPV5 URR I and HPV5 E2 expression vectors together with E1 expression vectors of HPV types -11, -16 or -18 and analysed replication of the origin plasmid. Our analysis showed that all combinations that we used supported replication, albeit at different efficiencies. HPV5 E1 protein was the most efficient *trans*-acting factor to support URR I replication, whereas HPV11 E1 was the least potent (Fig 6B). Quantification of the results showed that the combination of HPV5 E2 and HPV18 E1 resulted in around 50% and 80% less efficient replication at 24 to 48 and 72 to 96 hours after transfection respectively. HPV16 E1 was slightly less efficient than HPV18 E1at 24 and 48 hours, and similarly efficient at 72 and 96 hours. HPV11 E1 was almost inactive in supporting replication in this combination (Fig 6C). Similar results were obtained by quantifying SB signals if HPV5 wt URR was used as the origin bearing plasmid (Fig 6D). We also performed an experiment in which expression vectors coding for E2 proteins from different HPV types were used in combination with HPV5 URR I and the HPV18 E1K237A expression vector. As shown in Fig 6E, only HPV5 E2 in combination with HPV18 E1 was able to support replication of the HPV5 origin bearing plasmid (Fig 6E, lanes 13–16), whereas HPV11, -16 and -18 E2 expression vectors failed to do so.

One possible reason for the inability of E2 proteins derived from *alpha*-HPVs to support the replication of HPV5 URR I is that our *alpha*-HPV E2 expression construct renders E2 proteins non-functional. To exclude this possibility, we co-transfected the respective replication proteins together with their own URR plasmids into the U2OS cells. As shown in Fig 6F, all E1 and E2 proteins from HPV types -11, -16 and -18 were able to support replication from their own origin containing plasmid.

**Fig 7:**
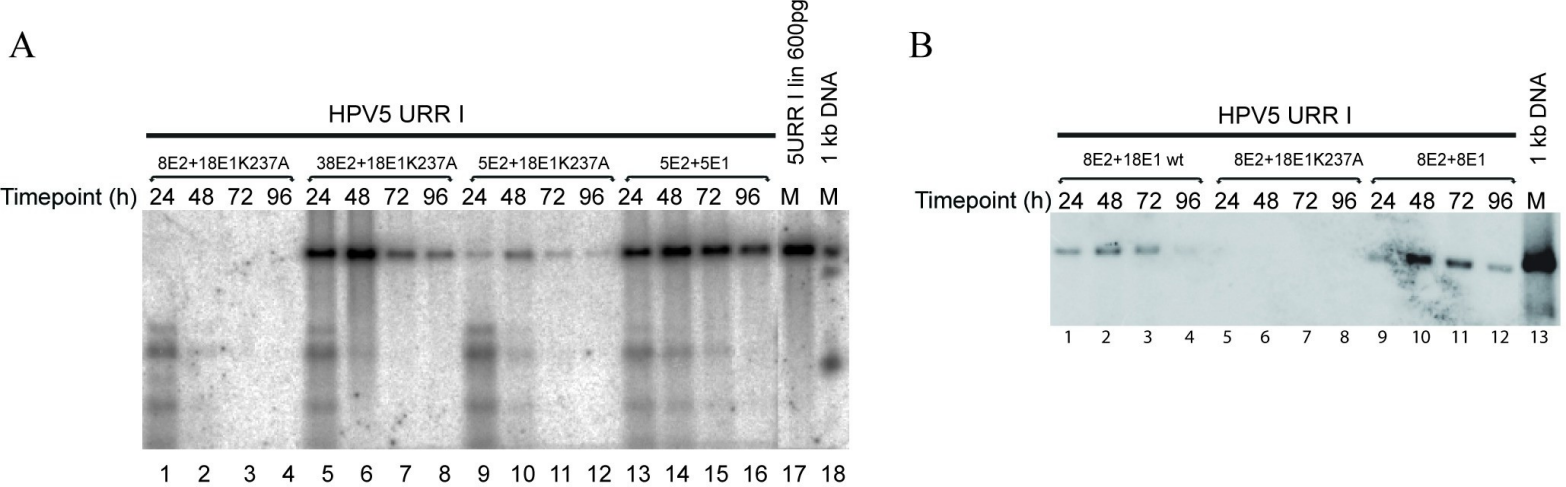
E2 proteins derived from *beta*-papillomaviruses HPV8 and HPV38 support replication of HPV5 URR I. (A) Replication of HPV5 E1BS minus origin URR I in the presence of HPV5 (lanes 9–12), -8 (lanes 1–4) and -38 (lanes 5–8) E2 expression vectors combined with HPV18 E1K237A expression vector. URR I combined with HPV5 E1 and E2 was used as a positive control (lanes 13–16). (B) Replication of HPV5 E1BS minus origin URR I with HPV8 E2 combined with HPV18 E1 wt (lanes 1–4) or E1K237A mutant (lanes 5–8). Replication of URR I with HPV8 E1 and E2 is used as a positive control (lanes 9–12). Total DNA was extracted at the indicated time points after transfection for both panels. DNA was digested with *DpnI* to remove input DNA, and an enzyme *(ScaI*) was employed to linearize the construct, which was resolved in agarose gel, and replication was analysed by SB.

Since the ability to support replication of HPV5 URR I lies in the properties of E2 protein, as E2 proteins from *alpha*-HPV types fail to do so, we asked if E2 proteins from other *beta*-HPVs support replication of HPV5 origin. By using expression vectors coding for HPV8 or -38 replication proteins, we showed that HPV8 E1 and E2 proteins are competent in supporting the replication of HPV5 URR I (Fig 3A, lanes 5–8), while HPV8 E2 in combination with HPV18 E1K237A is not (Fig 7A, lanes 1–4). However, HPV38 E2 together with HPV18 E1K237A can support replication from HPV5 URR I (Fig 7A, lanes 5–8).

The fact that HPV8 E2 protein was not able to support replication of HPV5 origin, if combined with the HPV18 E1K237A mutant, might mean that these proteins are completely incompatible with each other. However, as shown in Fig 6B and 6C, the E1K237A mutant is less potent than HPV18 wt E1 to support replication of HPV5 URR I. Therefore, it is possible that the combination of HPV18 E1 and HPV8 E2 will be functional if wt E1 is used. We tested this possibility and showed that wt E1 from HPV18 can support the replication of HPV5 URR I in combination with HPV8 E2 (Fig 7B, lanes 1–4).

**Fig 8:**
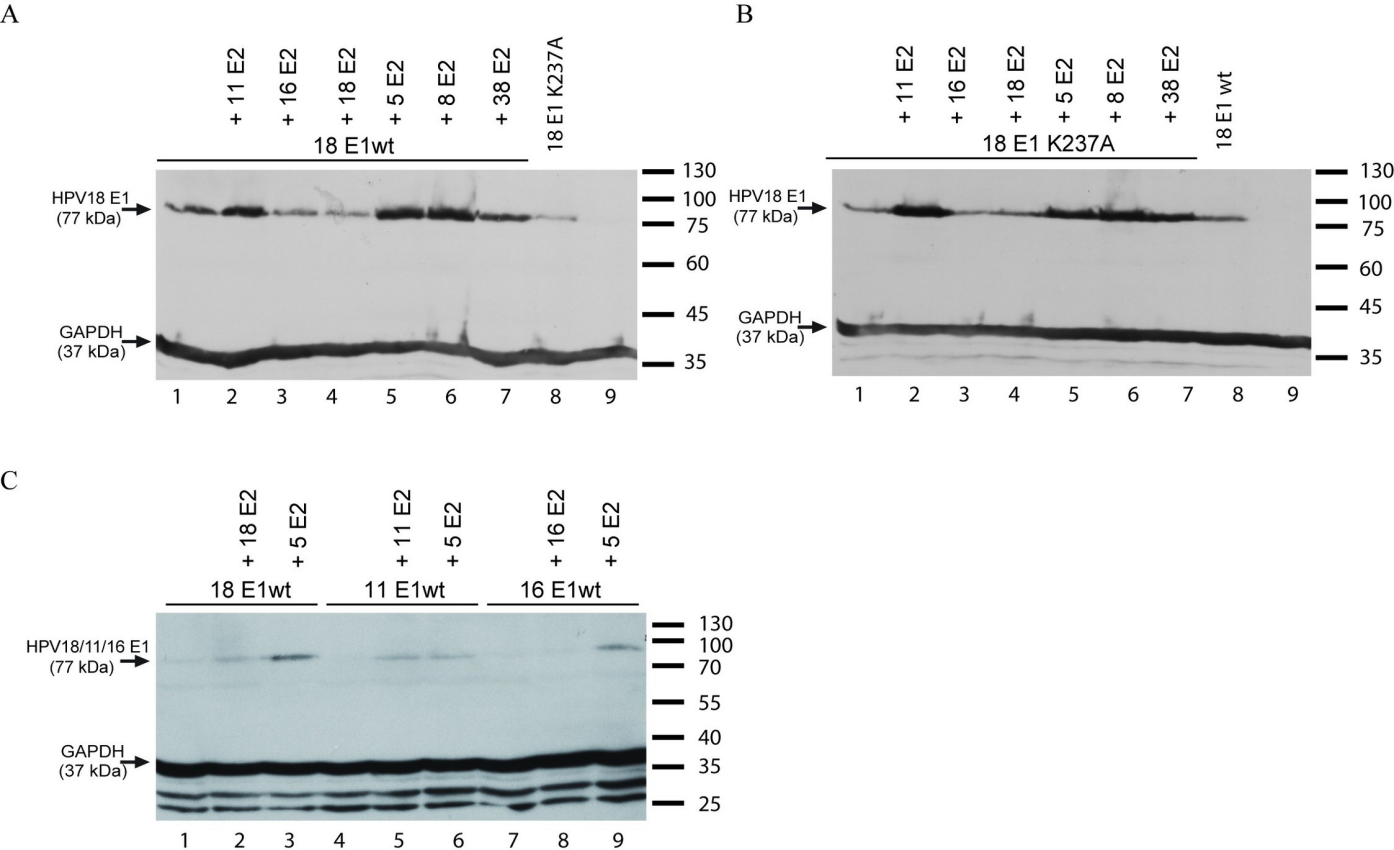
Steady-state levels of HPV11, -16, -18 E1 wt and -18 E1 mutant K237A proteins in the presence of *alpha*- and *beta*-PV E2 proteins. The expression vector coding for HA-tagged HPV11, -16, -18 E1 wt or -18 E1 K237A protein was transfected into U2OS cells either alone (A and B lanes 1, 8; C lanes 1, 4, 7) or together with the expression vectors of E2 proteins of *alpha*-PVs -11, -16 and -18 (A and B lanes 2 to 4; C lanes 2, 5, 8) or *beta*-PVs -5, -8 or -38 (A and B lanes 5 to 7; C lanes 3, 6, 9). (A, B) Protein extracts were made 48 h after transfection by lysing the cells in 120 μl 2x Laemmli buffer. Extracts were denatured, and 20 μl of protein was resolved by 10% SDS-PAGE. E1 levels were determined using anti-HA-tag antibody 16B12 (1:750). (C) RIPA extracts (80 μl) were made 48 h after transfection. Protein concentrations were measured, and 20 μg of protein extracts were resolved in 10% SDS-PAGE. Levels of GAPDH were used as loading controls.

One possible explanation for the inability of *alpha*-PV E2 proteins to support replication of HPV5 minimal origin is that E2 proteins from HPV5 and -8 can stabilize HPV18 E1, whereas E2 proteins from *alpha*-papillomaviruses fail to do so. To analyse this possibility, we performed a Western blot analysis and determined the levels of overexpressed HPV18 wt E1 and E1K237A protein in the presence of different *alpha-* and *beta-*PV E2 proteins. As shown in Fig 8A and 8B and S3 Fig, HPV18 E1 protein levels were higher if different *beta-*PV E2 proteins were present as compared to when HPV16 and 18 E2 expression vectors were used in co-transfections (Fig 8A and 8B, compare lanes 3–4 *alpha* E2 with 5–7 *beta* E2). HPV11 E2 was able to increase HPV18 E1 protein levels similarly to beta-PV E2 proteins (Fig 8A and 8B, lane 2). *Alpha*-PV (HPV11, 16) E1 protein levels were also higher in the presence of HPV5 E2 (Fig 8C, S3C Fig).

### E2 proteins from *alpha*-HPVs fail to support replication from *beta*-papillomavirus origins

**Fig 9:**
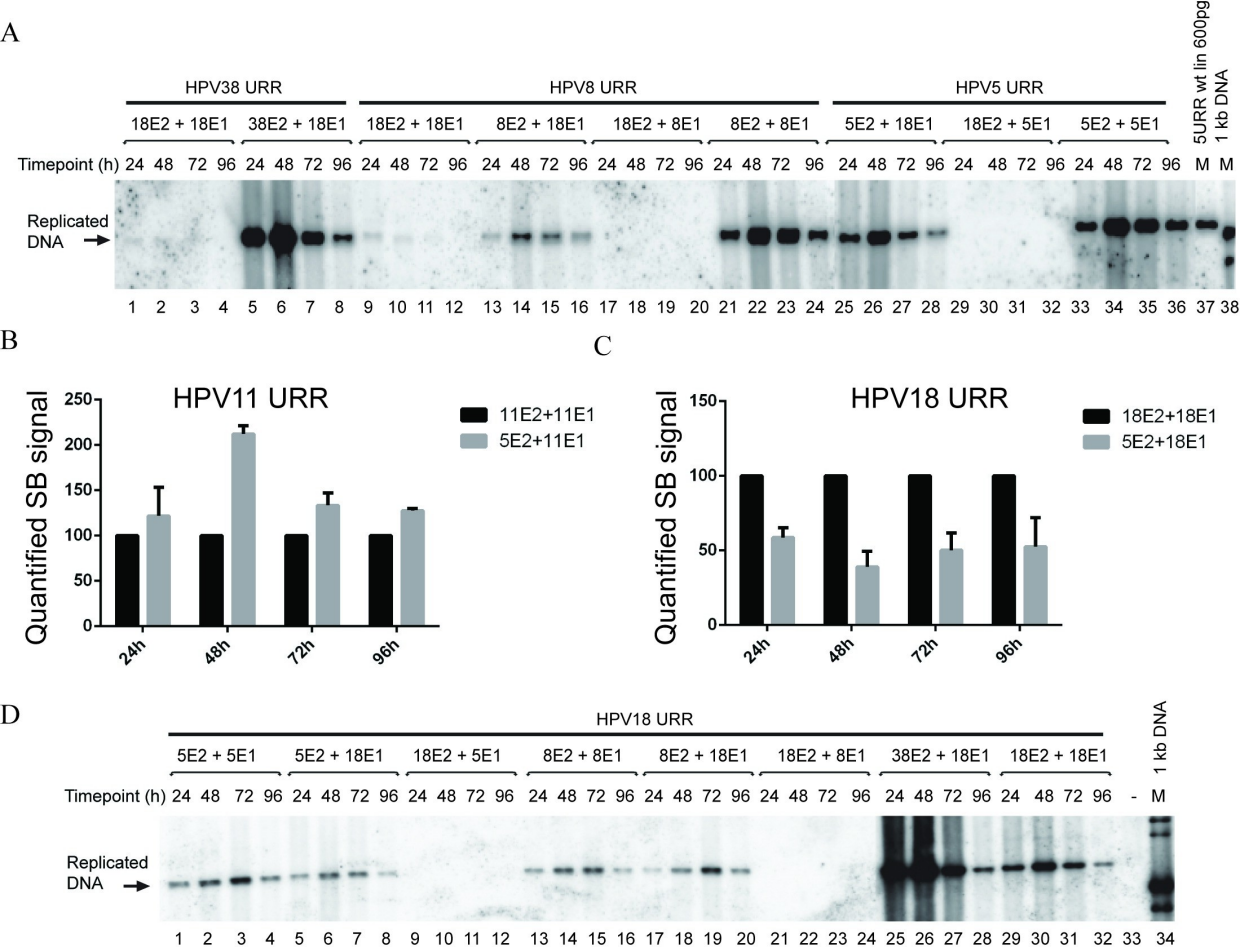
URR sequences from *alpha-* and *beta-*HPV types differ in their ability to replicate in the presence of viral trans factors from virus types of other genera. (A) Replication of *beta-*HPV URR sequences in combination with *beta-*HPV or HPV18 E2 and E1 proteins. U2OS cells were co-transfected with 500 ng HPV38 (lanes 1 to 8), -8 (lanes 9 to 24) or -5 (lanes 25 to 36) URR plasmids together with 250 ng HPV18 or HPV -5, -8 or -38 E2 expression vector and 100 ng HPV18,-5 or -8 E1 expression vectors. (B, C) Efficiency of the replication of *alpha*-HPV11 and -18 URR sequences in the presence of their own replication proteins, or HPV5 E2 and *alpha*-HPV E1 protein. U2OS cells were co-transfected with 500 ng URR plasmids together with 100 ng E1 and 250 ng E2 expression vectors coding for proteins from HPV types indicated in the figure. SB signals from three independent experiments were quantified and set as 100% for the HPV11 or -18 E1 and E2 combination. Data are presented as an average mean +/- SD. (D) Replication of HPV18 URR in the presence of *beta*-HPV E1 and E2 proteins. U2OS cells were co-transfected with 500 ng HPV18 URR plasmid together with 250 ng of HPV5, -8, -18 or -38 E2 expression vector and 100 ng of *alpha*-HPV18 E1 or *beta*-HPV5, -8 E1 expression vectors. HPV18 URR plasmid in combination with HPV18 E1 (100 ng) and E2 (250 ng) expression vectors (lanes 29 to 32) was used as a positive control. Total DNA was extracted at the indicated time points after transfection for all panels. DNA was digested with *DpnI* to remove input DNA, and an enzyme *(ScaI*) linearized the construct, which was resolved in agarose gel, and replication was analysed by SB.

Our previous results, showing that E2 proteins derived from *alpha*-papillomaviruses fail to support replication of HPV5 origin, were largely obtained using the HPV5 URR deletion construct (URR I), which lacks the E1 binding site, and some of the experiments were performed with the HPV18 E1K237A mutant. To corroborate our results and analyse whether this phenomenon is restricted to HPV5 origin, we used wt URR constructs of three different *beta*-HPVs (HPV5, -8, and -38). We combined these origins containing constructs with E1 and E2 expression vectors from HPV types -5, -8, -18 or -38 in co-transfection experiments. The results of this analysis are shown in Fig 9A. As we have shown previously using the HPV5 URR deletion construct URR I, the combinations of HPV5 E1 and E2 or HPV5 E2 with HPV18 E1 were able to support replication from HPV5 full-length URR, whereas the opposite combination of HPV18 E2 and HPV5 E1 failed to do so (Fig 9A, lanes 25–36). HPV38 URR was able to replicate in the presence of HPV38 E2 and HPV18 E1 but not when both viral proteins were derived from HPV18 (Fig 9A lanes 1–8). HPV8 URR replicated in the presence of HPV8 replication proteins or HPV8 E2 in combination with HPV18 E1 but not in the presence of E1 and E2 from HPV18 or with a combination of HPV8 E1 and HPV18 E2 (Fig 9A, lanes 9–24).

### URR sequences of *alpha*-HPV types replicate in the presence of *beta*-HPV E2 proteins but not with *beta*-HPV E1 proteins

To determine whether genus-specific E2 proteins are crucial for the replication of *alpha*-PV origins, we used HPV11, -16 and -18 origin plasmids and their respective E1 protein expression vectors and co-transfected these plasmids together with *beta-*PV type 5 E2 protein expression vector into the U2OS cells and analysed their replication capacity by SB (Fig 9B and 9C, S4A Fig).

HPV11 and HPV18 origins were replicating in the presence of HPV5 E2 protein, whereas HPV16 origin was not (Fig 9B and 9C, S4A Fig). HPV5 E2, together with HPV11E1 was able to support the replication of HPV11 URR at efficiencies comparable to HPV11 E1 and E2 combination (Fig 9B), whereas HPV5 E2 was clearly inferior in supporting the replication of HPV18 URR as compared to the HPV18 E2 protein (Fig 9C).

As our results showed that HPV5 E2 protein was able to support replication of *alpha*-HPV URRs, we investigated if E2 proteins from related HPV8 and -38 types behaved in a similar manner. We co-transfected HPV18 origin plasmid together with HPV5 or -8 E1 and HPV5, -8, 38 or HPV18 E2 protein expression vectors into U2OS cells and analysed replication by SB (Fig 9D). The results from these analyses showed that HPV18 URR was not replicating when HPV18 E2 and *beta-*PV -5 or -8 E1 proteins were used in combination (Fig 9D, lanes 9–12 and 21–24). Surprisingly, a high level of replication was observed when HPV18 URR was transfected together with HPV38 E2 and the HPV18 E1 expression vector (Fig 9D, lanes 25–28). The replication efficiency of this combination was substantially higher than HPV18 URR replication together with HPV18 E1 and E2 (Fig 9D, compare lanes 25–28 to 29–32). Quantification of the results showed that the combination of HPV38 E2 and HPV18 E1 supported the replication of HPV18URR up to seven times more efficiently than HPV18 E1 and E2 proteins (S4B Fig).

**Fig 10:**
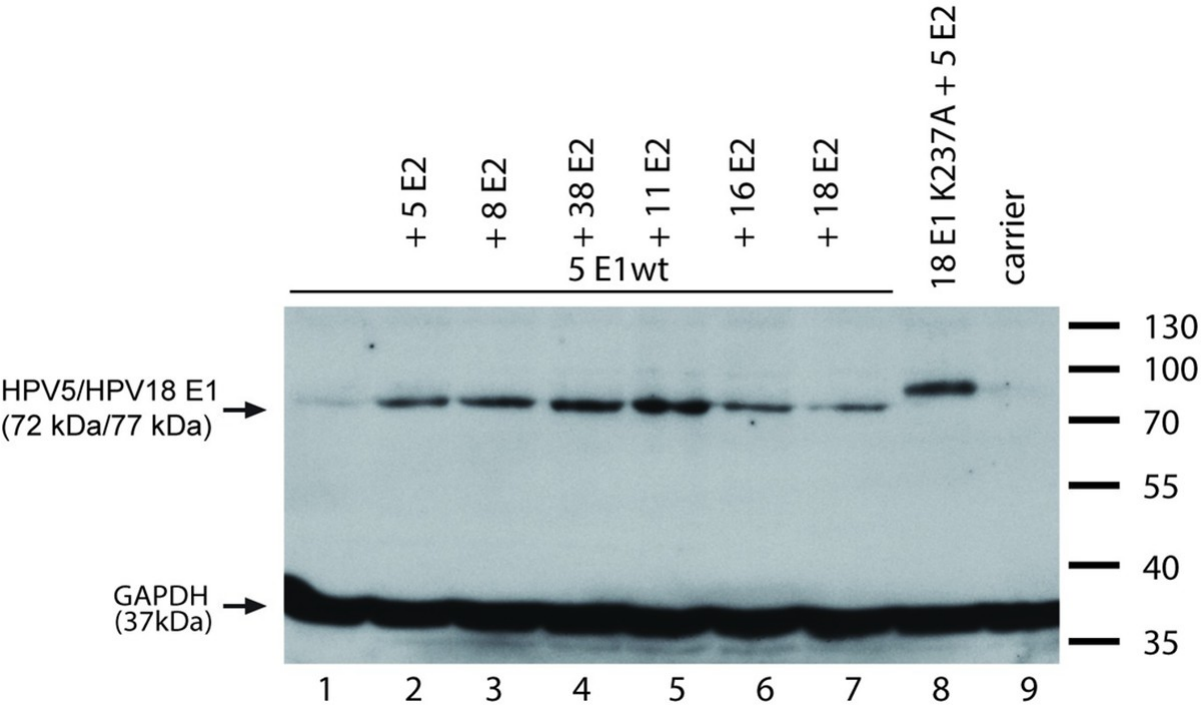
Steady-state levels of HPV5 E1 protein in the presence of *beta*- and *alpha*-PV E2 proteins. The expression vector coding for HA-tagged HPV5 E1 protein was transfected into U2OS cells either alone (lane 1) or together with the expression vectors of E2 proteins of *beta*-PVs -5, -8 or -38 (lanes 2 to 4) or *alpha*-PVs -11, -16 and -18 (lanes 5 to 7). Western blot analysis was performed 48 h after transfection using anti HA-tag antibody 16B12 (1:10 000). Levels of GAPDH were used as loading controls.

These results show that E1 proteins of *beta*-HPV types cannot cooperate with *alpha*-HPV E2 proteins to support replication of *alpha*-HPV URR plasmids. However, they can efficiently replicate the respective *alpha* origin-bearing plasmids when combined with *beta* E2 proteins (Fig 9D, lanes 1–4 and 13–16). Finally, we tested whether *alpha*-PV E2 proteins are able to increase the steady-state levels of HPV5 E1 protein by Western blot analysis, and we observed that all E2 proteins tested did so, including those from HPV11, -16 and -18 (Fig 10, S5 Fig).

Concise overview of the ability of all heterologous and homologous combinations of E1 and E2 proteins from *beta-* and *alpha-*HPV types to support replication of various HPV replication origins is shown in S2 Table.

## Discussion

### *Cis*-elements required for E1- and E2-dependent replication of HPV5 URR

Sequence requirements for the papillomavirus origin of replication have been studied extensively [16]. General conclusions drawn from these studies delineate PV origin as a tripartite DNA element consisting of binding sites for viral replication proteins E1 and E2 and an A/T-rich region. Closer inspection of these elements has shown, however, that not all of them are always essential for replication.

In the present article, we have studied the *cis*-sequence requirements for HPV5 origin of replication. We show that HPV5 URR can be divided into two halves, which are both capable of replication under permissive conditions in U2OS cells, albeit with lower efficiencies than the whole URR construct. The first half contains three E2 binding sites that are in their natural configuration. The second half contains an E1 binding site, one high-affinity E2 binding site, a low-affinity E2 binding site in the M29 region and an A/T-rich region. We further show that the A/T-rich region is also dispensable in this setting. However, if one deletes the M29 region, E1 binding site or both, then the origin becomes non-functional.

Several conclusions can be drawn from these results. First, it appears that E1- and E2-dependent HPV5 replication can be initiated at different positions inside the URR, since two almost non-overlapping regions of URR are able to replicate. Further studies are needed to investigate whether HPV5 replication is initiated from two distinct sites in the context of the viral genome. Second, our results show that the sequence corresponding to the E1 binding site in HPV5 URR is not absolutely necessary for the initiation of replication. It has been shown that E1 can bind DNA both specifically to its cognate site and non-specifically via its helicase domain [32][33][34]. It is plausible to speculate that if the E1 binding site is lacking in origin, the E2-mediated non-specific binding activity of E1 is sufficient to initiate replication.

Our conclusions are further strengthened by previous observations that synthetic constructs harbouring four E2 binding sites are able to replicate when HPV18 E1 and E2 are provided in *trans* [35]. It has also been shown previously that the E1 binding site can be mutated in the case of HPV11 and -18 origins, and such a sequence is able to replicate under permissive conditions [19]. In fact, the only papillomavirus type, where the requirement for E1 binding site for replication is convincingly demonstrated, is BPV1, which belongs to a distant *delta*-genera of papillomaviruses [1]. It is highly possible that different PV genera have different *cis* requirements to initiate replication and initiation of replication from *delta* papillomavirus origins requires an intact E1 binding site and sequence-specific E1 binding. Alternatively, it is plausible to speculate that the interaction between viral and host replication proteins is weaker in the inter-species replication system, and none of the studies with BPV1 are performed in calf cells, which is the natural host of the virus.

### Compatibility of E1 and E2 proteins from different genera in supporting replication of HPV URR sequences

It is known that replication of papillomavirus URR sequences can take place under conditions where expressed *trans*-elements E1 and E2 proteins are derived from another virus type. It has been established that BPV1 (*delta*-PV) and HPV11 (*alpha*-PV) E1 and E2 proteins can efficiently support the replication of *alpha*-PV (HPV6b, -7, 11, -16, -18), *kappa*-PV (CRPV), *delta*-PV (BPV1) and *mu*-PV (HPV1) URR containing plasmids [36][19]. HPV1a (*mu*-PV) E1 and E2 proteins can also support replication of *alpha*-PVs (HPV-6b and -18) and BPV1 origins of replication [37] [20]. These studies have led to the general consensus that the process of replication initiation is highly conserved within the papillomavirus family, and in principle, *trans*-factors and *cis*-elements required for this process are interchangeable.

Given that initiation of replication from HPV5 URR can take place at two distinct places along the sequence, we investigated if *trans*-factors (E1 and E2 proteins) from other HPV types can support this process. We show that within *beta*-PV genera, the factors required for initiation of replication seem to be fully compatible with each other both if HPV5 or -8 URR plasmids are used and if HPV5 URR I plasmid is used, which lacks E1 binding site. Surprisingly, E1 and E2 proteins from *alpha*-PV types HPV11, -16, and -18 do not support replication of the *beta*-PV URR-containing plasmids. In contrast, replication of the *alpha*-PV HPV18 URR-containing plasmid was supported by E1 and E2 proteins derived from *beta*-PV types HPV5 and -8. We further show that this inability of *alpha*-PV *trans-*factors to support *beta*-PV URR replication lies in the properties of viral E2 protein, since *alpha*-PV E1, in combination with *beta*-PV E2, is functional with all PV URRs tested.

Several reasons might account for the inability of *alpha*-PV E2 proteins to support replication of *beta*-PV URR sequences. First, it has been shown that E2 proteins stabilize E1 protein levels in cells. Therefore, it may well be that *alpha*-PV E2 proteins are unable to stabilize *beta*-PV E1 protein levels, and there is not enough E1 to initiate replication. We show that this inability is not the case, as HPV5 E1 protein levels are increased by all E2 proteins tested, including those from HPV11, -16 and -18.

Alternatively, it is possible that the nature of E2 binding sites determine the inability of *alpha*-PV E2 proteins to support *beta*-PV URR replication. The E2 binding sites of all viral strains consist of a highly conserved sequence, ACCGNNNNCGGT, where N is any base separating the palindromic half-sites [38] [39], [40] [41]. The 4-nucleotide spacer–NNNN–is conserved in length, but the sequence varies between species and individual binding site positions [42]. This linker sequence influences E2-binding affinity, although the protein does not make direct contacts with these nucleotides [43] [44]. According to previous studies, E2 proteins of *alpha*-PVs bind with higher affinity onto these E2 binding sites, which contain A/T nucleotides in the spacer, but E2 protein of *beta*-PVs onto these that have more G/C nucleotides in spacer [45] [46] [43][47] [48] [42]. The E2 binding sites in the HPV5, 8, -38 URR contain more G/C nucleotides in spacer than HPV11, -16, -18 E2BSs (S1B Fig); therefore, this might be one of the reasons why E2 and E1 proteins of *alpha*-papillomaviruses -11, -16 and -18 are not able to support replication of HPV5 origin. This hypothesis is also supported by the results showing that HPV5 wild-type and E1 binding site minus URR constructs are replicating in combination with HPV5 E2 protein and *alpha*-PVs wild-type E1 or E1 DNA binding activity minus protein. Other high-risk (HPV8) and low-risk (HPV38) *beta*-papillomavirus replication origins replicate in the combination of their E2 proteins and *alpha*-papillomavirus (HPV18) E1 protein. This finding indicates that the E2 proteins of *alpha*-PVs do not bind well to *beta*-PVs E2 binding sites. This weaker binding of *alpha*-PV E2 proteins onto *beta*-PV URR might also result in inappropriate bending of DNA, as it has been shown that E2 can bend DNA [45]. The inappropriate bending might in turn lead to a DNA configuration that is unfavourable for E1:E2 complex formation. Further studies are needed to verify this hypothesis.

Surprisingly, *beta*-PV E1 proteins in combination with HPV18 E2 protein do not support the replication of *alpha*-PV URR. In this case, the HPV18 E2 protein binds to E2BSs, and the *beta*-PV E1 and *alpha*-PV E2 protein complexes are also likely formed, as we observed from the Western blot assay that the HPV5 E1 protein levels are increased in the presence of *alpha*-PV E2 proteins. In this case, the defective replication activity might be due to the inappropriate conformation of the DNA-E2-E1 complex. This discrepancy might appear due to the difference in the length of the E2 protein hinge region, which is approximately three times shorter in *alpha*-PV E2 proteins than in *beta*-PV E2 proteins.

In conclusion, major differences exist in the *cis*-sequences of *alpha*- and *beta*-HPVs and the ability of viral replication proteins from different HPV genera to support replication of these *cis*-sequences. The general conclusion that can be drawn from this analysis indicates that *beta*-HPV E2 proteins are more potent in their ability to support replication of various HPV origins. This property of E2 proteins of *beta*-HPV types might be one of the reasons why cutaneous HPV genomes remain episomal more frequently compared to the genomes of mucosal HPV types, which often integrate into the host genome.

## Supporting information

S1 Fig Difference of *alpha*- and *beta*-papillomavirus URR sequences. (A) Schematic representation of *alpha*- (-11, -16, -18) and *beta*-(-5, -8, -38) papillomaviruses URRs. (B) Sequences of E2 and E1 binding sites in HPV5, -8, -11, -16, -18, -38 URRs.
(TIF)

S2 Fig Replication of HPV5 wt URR and E1BS minus origin in the presence of increasing amounts of E1 or E2 proteins. **(A) (B)** Replication of HPV5 wt URR (A) and URR I (B) in the presence of increasing amounts of HPV5 E1 protein. U2OS cells were co-transfected with 500 ng of the respective HPV5 URR construct together with 250 ng HPV5 E2 expression vector and increasing amounts of HPV5 E1 (from 10 ng to 250 ng) expression vector. **(C) (D)** Replication of HPV5 wt URR (C) and URR I (D) in the presence of increasing amounts of E2 protein. U2OS cells were co-transfected with 500 ng of the respective HPV5 URR construct together with 100 ng HPV5 E1 expression vector and increasing amounts of HPV5 E2 (from 10 ng to 250 ng) expression vector. Total DNA was extracted at the indicated time points after transfection for both panels. DNA was digested with *DpnI* to remove input DNA and an enzyme *(ScaI*) linearizing the construct, resolved in agarose gel, and replication was analysed by SB.
(TIF)

S3 Fig Quantification of the steady-state levels of HPV11, -16, -18 E1 wt and -18 E1 mutant K237A proteins in the presence of *alpha*- and *beta*-PV E2 proteins. Quantification of the E1 protein levels depicted in Fig 8. WB signals from three independent experiments were quantified and set as 1 for HPV18 E1 (A), HPV18 E1 K237A (B), HPV18 E1 (C, left panel), HPV11 E1 (C, middle panel) and HPV16 E1 (C, right panel). Data are presented as an average mean +/- SD.
(TIF)

S4 Fig Replication of *alpha*-PV URR sequences with different combinations of E1 and E2 proteins. (A) Replication of *alpha*-HPV URR sequences in the presence of HPV5 E2 and *alpha*-HPV E1 protein. U2OS cells were co-transfected with 500 ng URR plasmids together with 100 ng E1 and 250 ng E2 expression vectors coding for proteins from HPV types indicated in the figure. Total DNA was extracted at the indicated time points after transfection. DNA was digested with *DpnI* to remove input DNA, and an enzyme *(ScaI*) linearized the construct, which was resolved in agarose gel, and replication was analysed by SB. (B) Replication of HPV18 URR in the presence of *beta*-HPV E1 and E2 proteins. U2OS cells were co-transfected with 500 ng HPV18URR plasmid together with 250 ng of HPV5, -8, -18 or -38 E2 expression vector and 100 ng of *alpha*-HPV18 E1 or *beta*-HPV5, -8 E1 expression vectors. HPV18 URR plasmid in combination with HPV18 E1 (100 ng) and E2 (250 ng) expression vectors (lanes 29 to 32) was used as a positive control. SB signals from three independent experiments were quantified and set as 100% for the HPV18 E1 and E2 combination. Data are presented as an average mean +/- SD.
(TIF)

S5 Fig Steady-state levels of HPV5 E1 protein in the presence of *beta*- and *alpha*-PV E2 proteins. Quantification of the E1 protein levels depicted in Fig 10. WB signals from three independent experiments were quantified and set as 1 for HPV5 E1. Data are presented as an average mean +/- SD.
(TIF)

S1 Table Sequences of oligonucleotides. List of oligonucleotides and synthesized DNA fragments used in this study. Relevant restriction enzyme sites are shown in green.
(PDF)

S2 Table Abilities of heterologous and homologous combinations of E1 and E2 proteins from *beta*- and *alpha*-HPV types used in this study to support replication of various HPV replication origins. (PDF)
