# Supplementary figures and images for: E2 protein is the major determinant of specificity at the human papillomavirus origin of replication
Source: PLoS One. 2019 Oct 23;14(10):e0224334. doi: 10.1371/journal.pone.0224334 (PMC6808437; doi:10.1371/journal.pone.0224334)

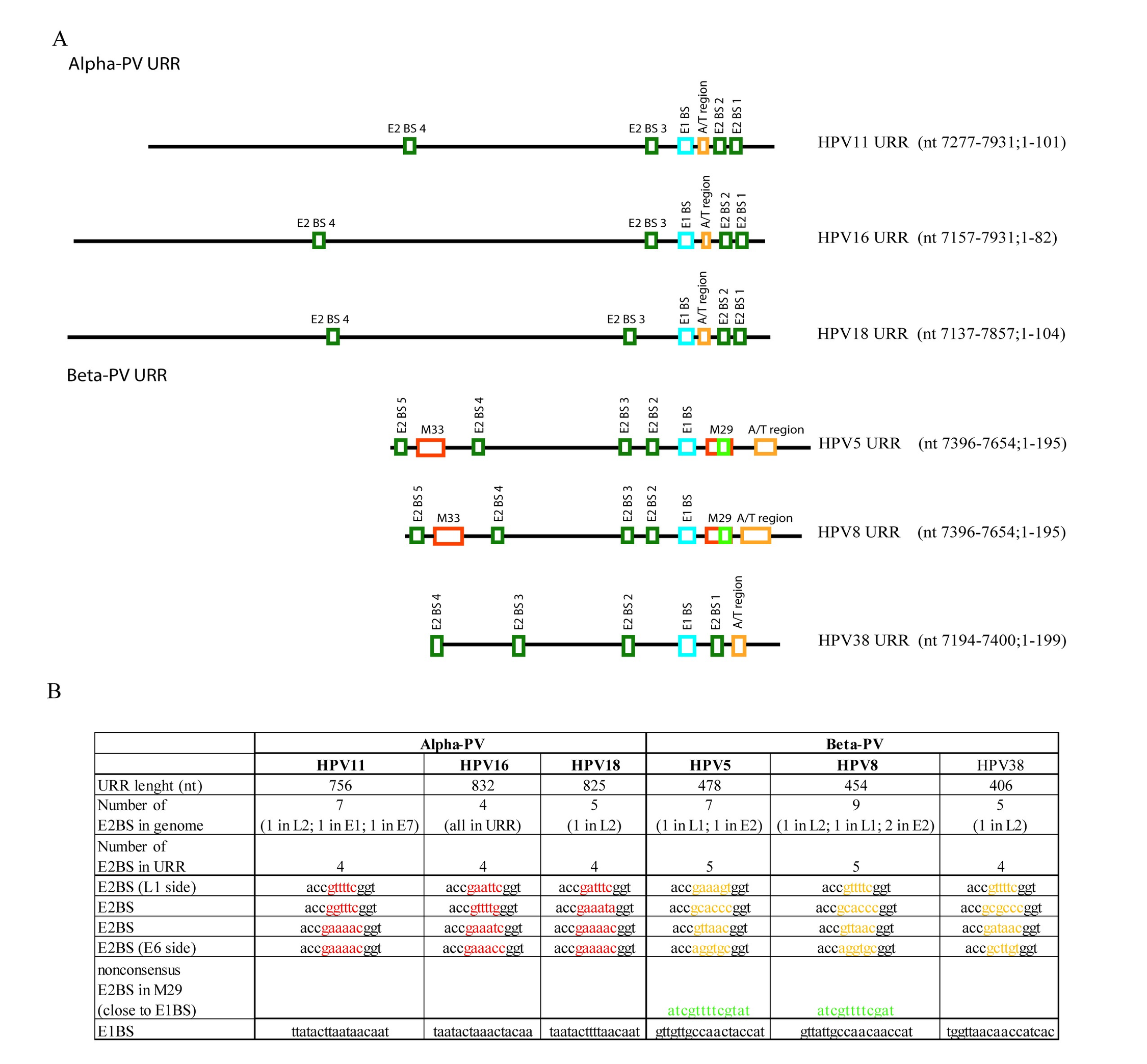

Supplement: S1 Fig — (A) Schematic representation of alpha- (-11, -16, -18) and beta-(-5, -8, -38) papillomaviruses URRs. (B) Sequences of E2 and E1 binding sites in HPV5, -8, -11, -16, -18, -38 URRs. (TIF) [file pone.0224334.s001.tif]

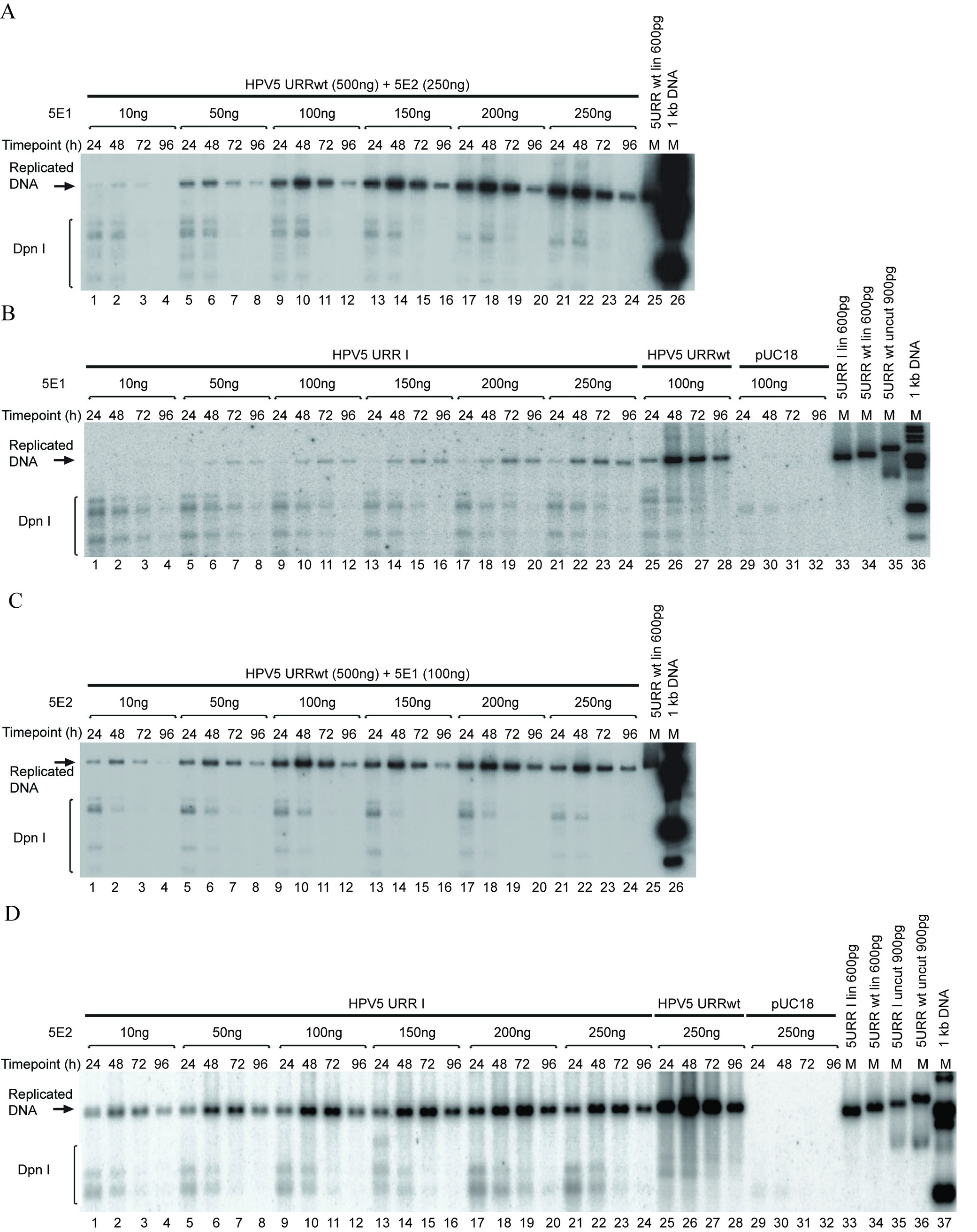

Supplement: S2 Fig — (A) (B) Replication of HPV5 wt URR (A) and URR I (B) in the presence of increasing amounts of HPV5 E1 protein. U2OS cells were co-transfected with 500 ng of the respective HPV5 URR construct together with 250 ng HPV5 E2 expression vector and increasing amounts of HPV5 E1 (from 10 ng to 250 ng) expression vector. (C) (D) Replication of HPV5 wt URR (C) and URR I (D) in the presence of increasing amounts of E2 protein. U2OS cells were co-transfected with 500 ng of the respective HPV5 URR construct together with 100 ng HPV5 E1 expression vector and increasing amounts of HPV5 E2 (from 10 ng to 250 ng) expression vector. Total DNA was extracted at the indicated time points after transfection for both panels. DNA was digested with DpnI to remove input DNA and an enzyme (ScaI) linearizing the construct, resolved in agarose gel, and replication was analysed by SB. (TIF) [file pone.0224334.s002.tif]

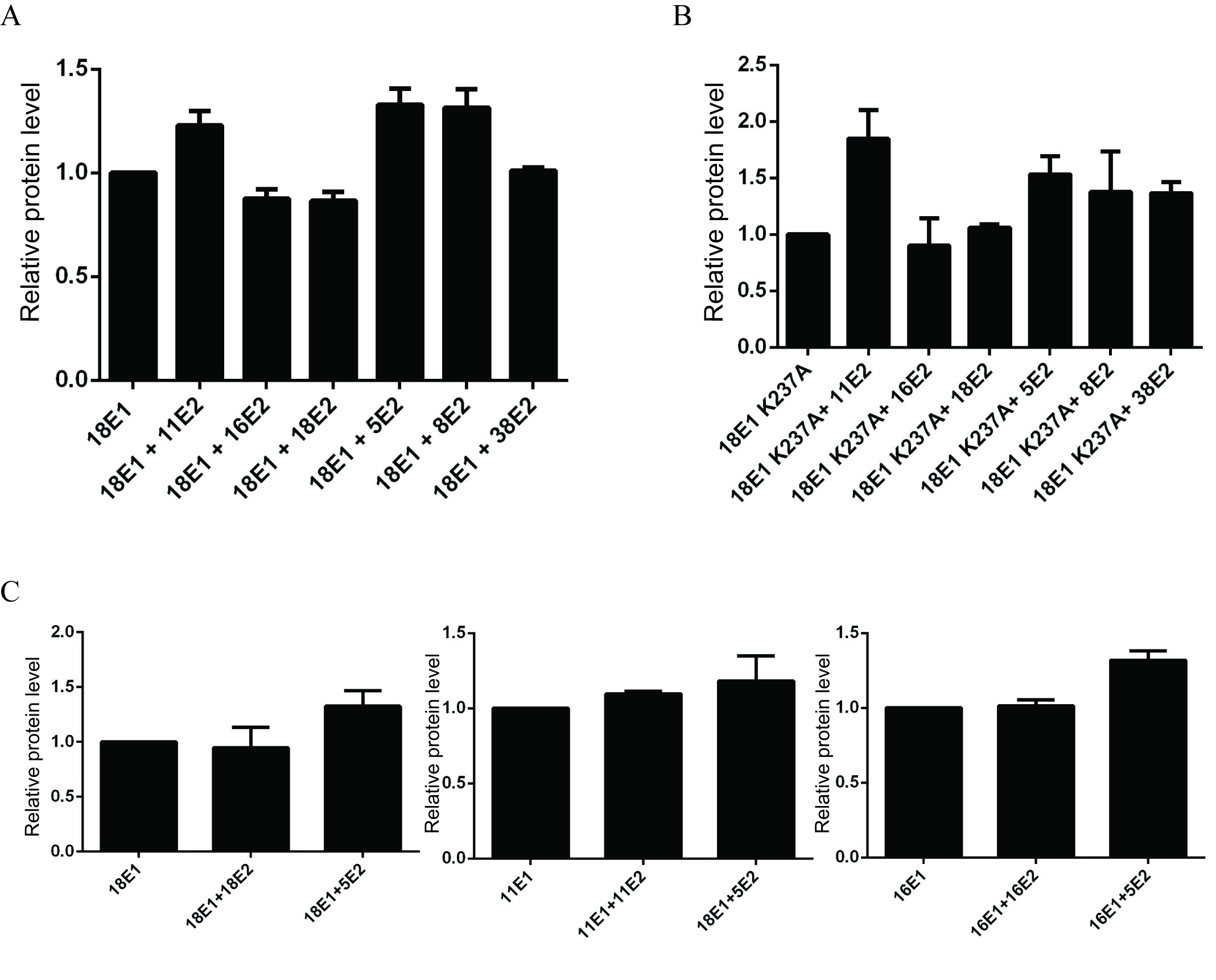

Supplement: S3 Fig — Quantification of the E1 protein levels depicted in Fig 8. WB signals from three independent experiments were quantified and set as 1 for HPV18 E1 (A), HPV18 E1 K237A (B), HPV18 E1 (C, left panel), HPV11 E1 (C, middle panel) and HPV16 E1 (C, right panel). Data are presented as an average mean +/- SD. (TIF) [file pone.0224334.s003.tif]

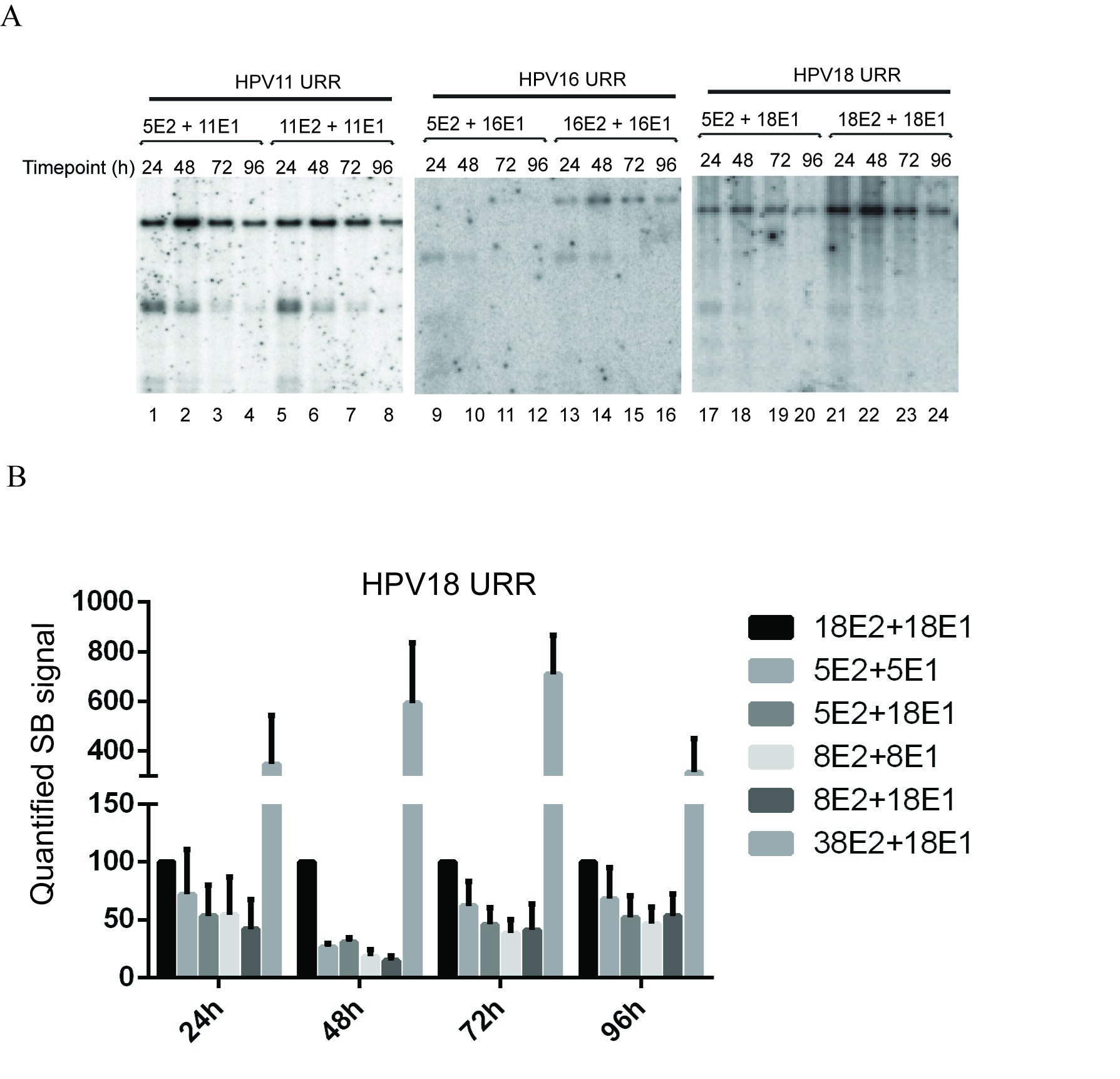

Supplement: S4 Fig — (A) Replication of alpha-HPV URR sequences in the presence of HPV5 E2 and alpha-HPV E1 protein. U2OS cells were co-transfected with 500 ng URR plasmids together with 100 ng E1 and 250 ng E2 expression vectors coding for proteins from HPV types indicated in the figure. Total DNA was extracted at the indicated time points after transfection. DNA was digested with DpnI to remove input DNA, and an enzyme (ScaI) linearized the construct, which was resolved in agarose gel, and replication was analysed by SB. (B) Replication of HPV18 URR in the presence of beta-HPV E1 and E2 proteins. U2OS cells were co-transfected with 500 ng HPV18URR plasmid together with 250 ng of HPV5, -8, -18 or -38 E2 expression vector and 100 ng of alpha-HPV18 E1 or beta-HPV5, -8 E1 expression vectors. HPV18 URR plasmid in combination with HPV18 E1 (100 ng) and E2 (250 ng) expression vectors (lanes 29 to 32) was used as a positive control. SB signals from three independent experiments were quantified and set as 100% for the HPV18 E1 and E2 combination. Data are presented as an average mean +/- SD. (TIF) [file pone.0224334.s004.tif]

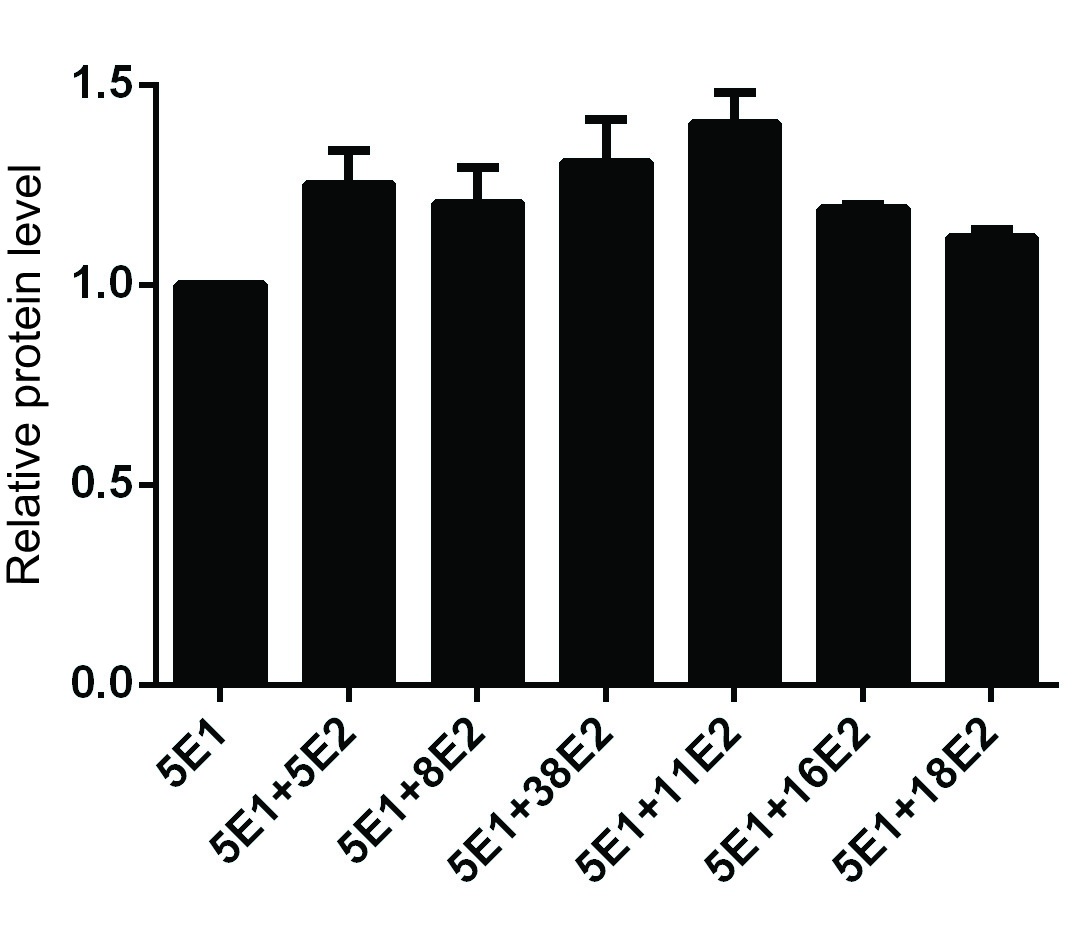

Supplement: S5 Fig — Quantification of the E1 protein levels depicted in Fig 10. WB signals from three independent experiments were quantified and set as 1 for HPV5 E1. Data are presented as an average mean +/- SD. (TIF) [file pone.0224334.s005.tif]
