# Supplementary material for: E2 protein is the major determinant of specificity at the human papillomavirus origin of replication
Source: PLoS One. 2019 Oct 23;14(10):e0224334. doi: 10.1371/journal.pone.0224334 (PMC6808437; doi:10.1371/journal.pone.0224334)
Supplement: S1 Table — List of oligonucleotides and synthesized DNA fragments used in this study. Relevant restriction enzyme sites are shown in green. (PDF) [file pone.0224334.s006.pdf]

Nr	Oligonucleotide	Sequence
**pUC18_URR**		
1	PrHPV5URR_XbaI_F	ATTCTAGAGGTCTGACCGAAAGTGG
2	PrHPV5URR_EcoRI_R	ATGAATTCTGCCTGATTACAGAAGAAC
3	PrHPV5URRI_EcoRI_R	ACCGAATTCACAAGAAAATTGC
4	PrHPV5URRII_XbaI_F	ATTTCTAGAGTTGCAATTTTCTTG
5	PrHPV5URRIII_EcoRI_R	ACCGAATTCTATTGGATATTGTTAACA
6	HPV5URR_IV	5`ACGACCGTTAACGGTAAGTTGCAATTTTCTTGTACCAGGTGCGGTATTAA GATTTCAC 3´<br>5´GTGAAATCTTAATACCGCACCTGGTACAAGAAAATTGCAACTTACCGTT AACGGTCGT 3´
7	HPV5URR_V	5`CTTGTACCAGGTGCGGTATTAAGATTTCACAATTGTAATGGTTGTTGCCA ACTACCATAGGCATA 3´<br>5´TATGCCTATGGTAGTTGGCAACAACCATTACAATTGTGAAATCTTAATAC CGCACCTGGTACAAG 3´
8	PrHPV8URR_XbaI_F	ATTCTAGAATTGTACCGTTTTCGGTAC
9	PrHPV8URR_EcoRI_R	ATGAATTCTTGCTTAGGAAAATTGCAC
10	PrHPV38URR_XbaI_F	ATTCTAGACCGTTTTCGGTCGCCC
11	PrHPV38URR_EcoRI_R	ATGAATTCGAATACATCCCAAGCTTACAG
**pQMN-TAi-_E1**		
12	PrHPV5E1_XbaI_F	GGTCTAGAATGACGGGATCCTAATTCTAAAG
13	PrHPV5E1_XmaI_R	ATCCCGGGTCATAAATGTTCATTAGCTG
14	HPV5 E1	5´CCTTCTAGAGCTGAGGGAGCCGAACACCAACCAAAACTGACAGAAAAA GATAAGGCAGAATTTCCTTCAAGTATTAGAGAGTTAGCTGAAACCTTAGGC ATCCCTCTGATTGATTGTTTAATACCTTGCAATTTCTGTGGCAAATTTCTAA ATTATTTGGAAGCTTGCGAATTCGACTACAAAAAACTTAGTCTAATTTGGA AAGATTATTGTGTGTTTGCGTGCTGTCGCGTATGCTGTGGCGCCACTGCAA CTTATGAATTTAACCAATTTTATGAGCAGACAGTTTTAGGACGAGATATTG AGTTAGCTTCAGGACTTTCGATTTTTGATATTGATATCAGGTGTCAAACTTG CTTAGCATTTCTTGACATTATAGAAAAGTTAGATTGCTGTGGCAGAGGCCT TCCCTTTCATAAGGTGAGGAACGCCTGGAAGGGAATCTGTAGGCAGTGTA AGCATTTTTATCACGATTGGTAAAGAGGTCACCGTGCAAGATATTATTCTG GAGCTCAGTGAGGTGCAGCCCGAAGTGCTACCAGTTGACCTGTTTTGTGA AGAGGAATTACCAAACGAGCAGGAAACGGAGGAGGAGCCTGACAACGAA AGGATCTCTTACAAAGTTATAGCTCCGTGCGGTTGCAGGAACTGTGAGGTC AAGCTTCGCATTTTTGTCCACGCCACAGAATTTGGTATTAGAGCTTTCCAA CAGCTACTGACCGGAGATCTGCAGCTCCTGTGCCCCGACTGTCGCGGAAA CTGCAAACATGACGGATCCTAATTACCCATACGATGTTCCAGATTACGCT AGCTCTAAAGGTAGTACATCTAAAGAAGGGTTTGGTGATTGGTGTTTATTG GAAGCTGACTGTAGTGATGTAGAAAATGATTTGGGACAATTATTTGAGAGA GATACAGACTCTGATATATCGGATTTGTTAGATGATACTGAACTGGAGCAG GGCAATTCTTTGGAACTATTTCATCAACAGGAGTGTGAGCAGAGCGAGGA GCAATTACAAAAACTAAAACGAAAGTATCTTAGTCCAAAAGCTGTCGCAC AGCTTAGTCCGCGACTTGAGTCAATT 3´
15	PrHPV8E1_HA_F	GGCGTCTCATGTTCCAGATTACGCTAGCGGTAGTACATCTAAAGAAGGG
16	PrHPV8E6_XbaI_F-ATG	ATTCTAGAGACGGGCAGGACAAGG
17	PrHPV8E7_HA_R	GGCGTCTCGAACATCGTATGGGTATTTATGATCCGCCATGTTTG
**pQMN-TAi-_E2**		
18	PrHPV5E2_BamHI_F	ATGGATCCGAGAATCTCAGCGAGCG
19	PrHPV5E2_HindIII_R	ATAAAGCTTTTAAAGACTGTCCAGGTTG
20	PrHPV8E2_BamHI_F	TAGGATCCGAGAATCTCAGCGAGCG
21	PrHPV8E2_HindIII_R	ATTAAGCTTTTATAGACTGTCCAGG
22	PrHPV38E2_HindIII_F	ATTAAGCTTATGGAAACTCTCAGCGC
23	PrHPV38E2_XmaI_R	TTCCCGGGCTATAAATCATCAAATTGTCC
