# Supplementary material for: E2 protein is the major determinant of specificity at the human papillomavirus origin of replication
Source: PLoS One. 2019 Oct 23;14(10):e0224334. doi: 10.1371/journal.pone.0224334 (PMC6808437; doi:10.1371/journal.pone.0224334)
Supplement: S2 Table — (PDF) [file pone.0224334.s007.pdf]

***beta*-HPV replication**							
**HPV5 URR wt**		**E1**					
		β-HPV 5	*β*-HPV 8	*α*-HPV 18	*α*-HPV 11	*α*-HPV 16	HPV18 E1 DBD-
**E2**	HPV5	+	+	+	+	+	+
	HPV8	+	+	ND	ND	ND	ND
	HPV18	-	ND	-	ND	ND	-
	HPV11	ND	ND	ND	-	ND	ND
	HPV16	ND	ND	ND	ND	-	ND
**HPV5 URR E1DBS- (URR I)**		*β*-HPV 5	*β*-HPV 8	*α*-HPV 18	*α*-HPV 11	*α*-HPV 16	HPV18 E1 DBD-
**E2**	HPV5	+	+	+	+	+	+
	HPV8	+	+	+	ND	ND	-
	HPV38	ND	ND	ND	ND	ND	+
	HPV18	ND	ND	-	ND	ND	-
	HPV11	ND	ND	ND	-	ND	-
	HPV16	ND	ND	ND	ND	-	-
**HPV8 URR wt**		*β*-HPV 5	*β*-HPV 8	*α*-HPV 18			
**E2**	HPV5	+	+	ND			
	HPV8	+	+	+			
	HPV18	ND	-	-			
**HPV38 URR wt**		*β*-HPV 8	*α*-HPV 18				
**E2**	HPV38	+	+				
	HPV18	ND	-				
***alpha*-HPV replication**							
**HPV18 URR wt**		*β*-HPV 5	*β*-HPV 8	*α*-HPV 18			
**E2**	HPV5	+	ND	+			
	HPV8	ND	+	+			
	HPV38	ND	ND	+			
	HPV18	-	-	+			
**HPV16 URR wt**		*α*-HPV 16					
**E2**	HPV5	+					
	HPV16	+					
**HPV11 URR wt**		*α*-HPV 11					
**E2**	HPV5	+					
	HPV11	+					

*ND – no data
